# Conformational dynamics modulate the catalytic activity of the molecular chaperone Hsp90

**DOI:** 10.1038/s41467-020-15050-0

**Published:** 2020-03-16

**Authors:** Sophie L. Mader, Abraham Lopez, Jannis Lawatscheck, Qi Luo, Daniel A. Rutz, Ana P. Gamiz-Hernandez, Michael Sattler, Johannes Buchner, Ville R. I. Kaila

**Affiliations:** 1 Center for Integrated Protein Science Munich at the Department of Chemistry, Technical University of Munich, Lichtenbergstrasse 4, D85748 Garching, Germany; 2 Institute of Structural Biology, Helmholtz Zentrum München, Ingolstädter Landstrasse 1, Neuherberg, 85764 Germany; 3 Soft Matter Research Center and Department of Chemistry, Zhejiang University, Hangzhou, 310027 China; 4 Department of Biochemistry and Biophysics, Stockholm University, SE-10691 Stockholm, Sweden

**Keywords:** Enzyme mechanisms, Chaperones, Molecular modelling, NMR spectroscopy, SAXS

## Abstract

The heat shock protein 90 (Hsp90) is a molecular chaperone that employs the free energy of ATP hydrolysis to control the folding and activation of several client proteins in the eukaryotic cell. To elucidate how the local ATPase reaction in the active site couples to the global conformational dynamics of Hsp90, we integrate here large-scale molecular simulations with biophysical experiments. We show that the conformational switching of conserved ion pairs between the N-terminal domain, harbouring the active site, and the middle domain strongly modulates the catalytic barrier of the ATP-hydrolysis reaction by electrostatic forces. Our combined findings provide a mechanistic model for the coupling between catalysis and protein dynamics in Hsp90, and show how long-range coupling effects can modulate enzymatic activity.

## Abstract (web summary)

The chaperone Hsp90 uses the free energy from ATP hydrolysis to control the folding of client proteins in eukaryotic cells. Here the authors provide mechanistic insights into how its catalytic activity is coupled to conformational changes by combining large-scale molecular simulations with NMR, FRET and SAXS experiments.

## Introduction

**Fig. 1:**
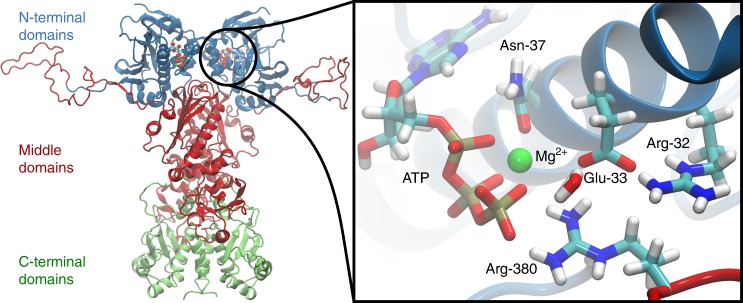
Structure of the yeast Hsp90 dimer (PDB ID: 2CG9)^9^. The N-terminal domains of Hsp90 are shown in blue, the middle domains in red, and the C-terminal domains in green. The inset shows a structure of the active site with a bound ATP molecule obtained from an MD simulation, where Asn-37 undergoes a rotation to form a stronger coordination to the magnesium.

The heat shock protein 90 (Hsp90) is an ATP-dependent molecular chaperone that controls protein maturation and folding, in addition to central regulatory functions of the eukaryotic cell^1–3^. Stringent clients of Hsp90 include 60% of the human kinome and the chaperone is therefore of significant biomedical interest. This 90 kDa, highly conserved, homodimeric enzyme comprises an N-terminal domain (NTD), a middle domain (M-domain), and a C-terminal domain (CTD) (Fig. 1)^4^. During its chaperone cycle, Hsp90 undergoes large-scale conformational changes from an open inactive state to a closed active state, which induce association of the M-domains and NTDs^5,6^. The closing exposes important client-binding regions, as shown in recent cryo-EM structures of Hsp90 in complex with co-chaperones and clients^7,8^. The ATP-binding site of Hsp90 is located in the NTD, and it is completed by residues from the M-domain, including an arginine (Arg-380 in yeast Hsp90) that forms contacts with the γ-phosphate of ATP^9^. ATP-binding triggers large conformational changes in the global Hsp90 structure, stabilizing the compact closed conformation of the enzyme^9^.

The thermodynamic driving force for this global conformational change originates from the tightly coupled slow ATPase reaction on *~*0.1–1 min^−1^ timescales^10^. Elnatan et al.^11^ further showed that ATP hydrolysis in the two protomers is sequential and deterministic in the mitochondrial Hsp90, TRAP1, with different hydrolysis rates for each protomer. After the first hydrolysis step, the Hsp90 dimer was suggested to undergo a flip into a closed asymmetric state, followed by a second hydrolysis event. Site-directed mutagenesis experiments identified several residues involved in the ATPase reaction: Glu-33 of the yeast Hsp90 is important for the ATP-hydrolysis reaction, as the E33A variant is able to bind ATP, but not to hydrolyse it^10,12,13^. Moreover, the R380A variant has a substantially lowered ATPase activity in comparison with the wild-type (WT) enzyme^4,13^ and N37A prevents Hsp90 from binding ATP due to loss of the catalytically important Mg^2+^ ion (Fig. 1)^12^. Furthermore, as for many other ATPases^14–16^, a water molecule is required for the ATP-hydrolysis reaction^9^. However, despite enormous efforts to study Hsp90 by structural, biochemical, biophysical, and computational approaches in the last decades^17–22^, the specific functions as well as the overall catalytic mechanism and its coupling to the conformational state of Hsp90 still remain unclear.

To probe the energetics, dynamics, and molecular mechanism of the ATP-hydrolysis reaction and its coupling to the conformational changes in Hsp90, we integrate here multi-scale computational simulations with biophysical experiments. We probe the coupling between the conformational dynamics of Hsp90 and its catalytic mechanism using hybrid quantum/classical (QM/MM) free-energy calculations in combination with large-scale atomistic molecular dynamics (MD) simulations. Our mechanistic models are further probed by site-directed mutagenesis experiments in combination with Förster-resonance energy transfer and nuclear magnetic resonance (NMR) spectroscopy, as well as by small-angle X-ray scattering (SAXS) measurements.

## Results

### Catalytic mechanism of ATP hydrolysis in Hsp90

To study the bond-formation/bond-breaking energetics during catalysis within the active site of Hsp90, we created hybrid QM/MM models, where the electronic structure of the chemically reacting system is computed on-the-fly, based on quantum mechanical density functional theory (DFT), and polarized by a classical force field model of the remaining protein framework. These QM/MM calculations were used to predict a free-energy surface for the ATP-hydrolysis reaction, by dynamically sampling conformational changes within the protein during the chemical transformation (Supplementary Fig. 1).

**Fig. 2:**
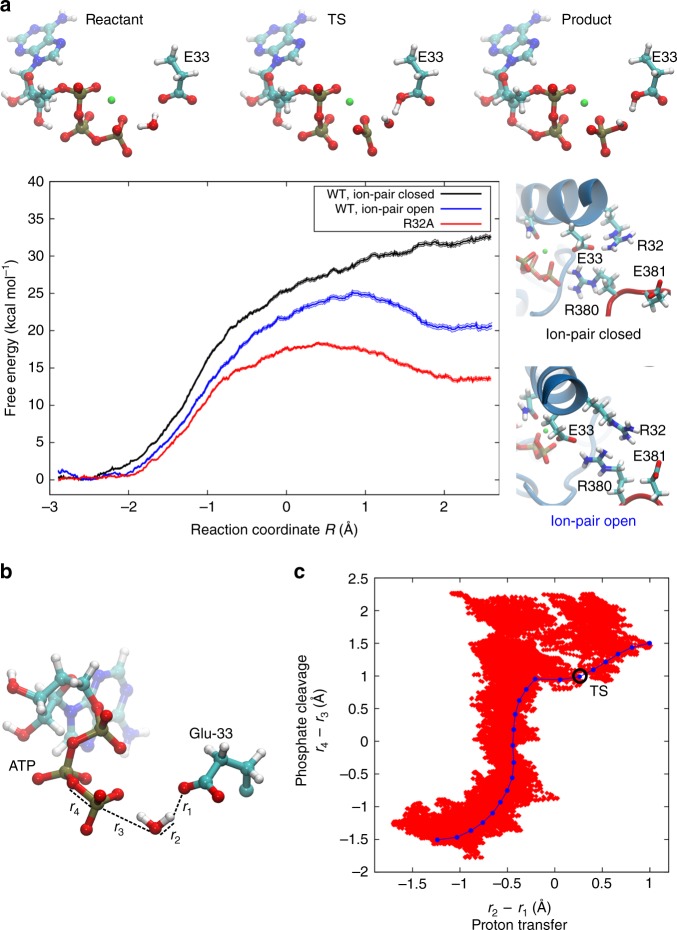
Energetics and dynamics of ATP hydrolysis in Hsp90. **a** Reactant, transition state (TS), and product structures showing ATP, Mg^2+^ (in green), and the sidechain of Glu-33 (E33), extracted from QM/MM calculations of the ATP-hydrolysis reaction. QM/MM free-energy profiles were calculated with the Arg-32/Glu-33 ion pair closed (black, *r*_R32-E33_ < 5 Å) and open (blue, *r*_R32-E33_ > 5 Å, see Fig. 3), as well as for the R32A mutant (red). **b** The reaction coordinate used for QM/MM calculations is *R* = *r*_4_ − *r*_3_ + *r*_2_ − *r*_1_, a linear combination of distances between Glu-33, the attacking water molecule, and the γ-phosphate of ATP. *r*_2_ – *r*_1_ is the difference between the bond-breaking (*r*_2_) and bond forming (*r*_1_) distances for the p*r*oton transfer from the water molecule to Glu-33. *r*_4_ – *r*_3_ is the difference between the bond-breaking (*r*_4_) and bond forming (*r*_3_) distances for the phosphate cleavage^27, 28^. The reaction coordinate was optimized from reactants (*R* = −2.9 Å) to products (*R* = 2.6 Å). **c** Semi-concerted ATP-hydrolysis mechanism from QM/MM free-energy calculations (red dots) and during reaction path optimization (blue dots), showing the sampled reaction coordinates with the Arg-32/Glu-33 ion pair closed. The transition state (TS) of the reaction path optimization is marked with a black circle.

Our QM/MM free-energy calculations suggest that the ATP-hydrolysis reaction is initiated by proton transfer between a water molecule, coordinating the ATP γ-phosphate, and Glu-33 (Fig. 2a, b). This water molecule is not present in the resolved X-ray structures of Hsp90^9^, but it originates from the bulk water by spontaneously coordinating between the γ-phosphate and Glu-33 during the atomistic MD simulations. After the water deprotonation reaction, the transient OH^−^ species immediately attacks the γ-phosphate, resulting in the cleavage of the β/γ-phosphate bond. The computed reaction free-energy profiles predict that the proton transfer and phosphate cleavage processes take place semi-concertedly rather than by associative or dissociative reaction steps (Fig. 2c)^23^. The calculations also suggest that the transition state comprises a penta-coordinated γ-phosphate, with elongated axial bonds, resembling the transition states found in other ATPases (Fig. 2a)^24–26^.

Interestingly, despite the chemically feasible reaction intermediates, the QM/MM calculations performed on a model of Hsp90 extracted from the experimental X-ray structure, suggest that the free-energy barrier for the hydrolysis reaction is >30 kcal mol^−1^ (Fig. 2a black trace, Supplementary Fig. 2, and Supplementary Table 1), which implies that the reaction rate is kinetically inaccessible (see Methods). Similar reaction energetics are obtained using different DFT approximations in the quantum chemical calculations, suggesting that the results are robust (Supplementary Table 2). Endergonic free-energy profiles have also been obtained in other QM/MM studies on phosphate cleavage processes^27,28^, whereas entropic effects are likely to stabilize the product state upon P_i_ dissociation up to ca. 17 kcal mol^−1^ (Supplementary Table 3), which could be transduced into driving conformational changes in Hsp90. Moreover, the free-energy profile does not show a clear minimum for the product state, indicating that the crystal structure may not represent a hydrolytically fully active state.

**Fig. 3:**
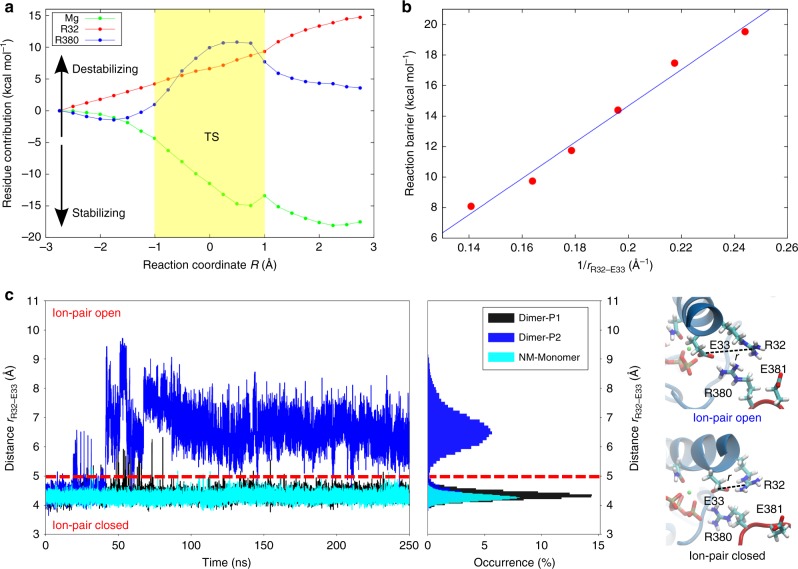
Analysis of the catalytic effects in Hsp90. **a** Energy decomposition of the catalytic effects. The figure shows the energy difference relative to the wild-type calculation, obtained by switching off individual residue contributions, with the transition state (TS) region marked in yellow (see also Supplementary Fig. 3). **b** Reaction barrier for ATP hydrolysis at different Arg-32/Glu-33 distances (see also Supplementary Fig. 4). The energy barrier is ca. 20 kcal mol^−1^ in the DFT models and thus slightly lower as compared with the QM/MM models with explicit treatment of the protein surroundings. **c** Arg-32/Glu-33 distance from MD simulations of the full-length Hsp90 dimer (in blue and black) and for the monomeric NM-domain model of Hsp90 (in cyan). All Arg-32/Glu-33 distances are measured between the Cζ (Arg-32) and Cδ (Glu-33).

To elucidate the molecular basis for this high barrier, we decomposed the energetic contributions of individual residues by switching off their interactions with the active site region during the calculations (Fig. 3a and Supplementary Fig. 3). These calculations suggest that the high reaction barrier associated with the proton transfer reaction originates from the low proton affinity of Glu-33, which in turn, results from the stabilizing interaction with the nearby Arg-32, favouring the deprotonated form of the glutamate. Calculations based on DFT models show that the reaction barrier has a coulombic 1/distance–dependence on the Arg-32/Glu-33 sidechain distance, suggesting that the reaction is electrostatically tuned (Fig. 3b and Supplementary Fig. 4). The analysis further shows that the Mg^2+^ ion pulls away electron density from the γ-phosphate, lowering the reaction barrier, whereas Arg-380 stabilizes the OH^−^ group attacking the γ-phosphate, as well as the resulting phosphate (HPO_4_^2−^) product (Fig. 3a, negative curvature for the R380 profile at *R* < −2.5 Å and *R* > 1 Å). Arg-380 could thus be central for stabilizing the transient hydroxide species, but is not directly involved in the water deprotonation reaction. These findings are consistent with the kinetically slow but viable R380A mutant^29^.

### Protein conformational changes modulate catalytic activity

**Fig. 4:**
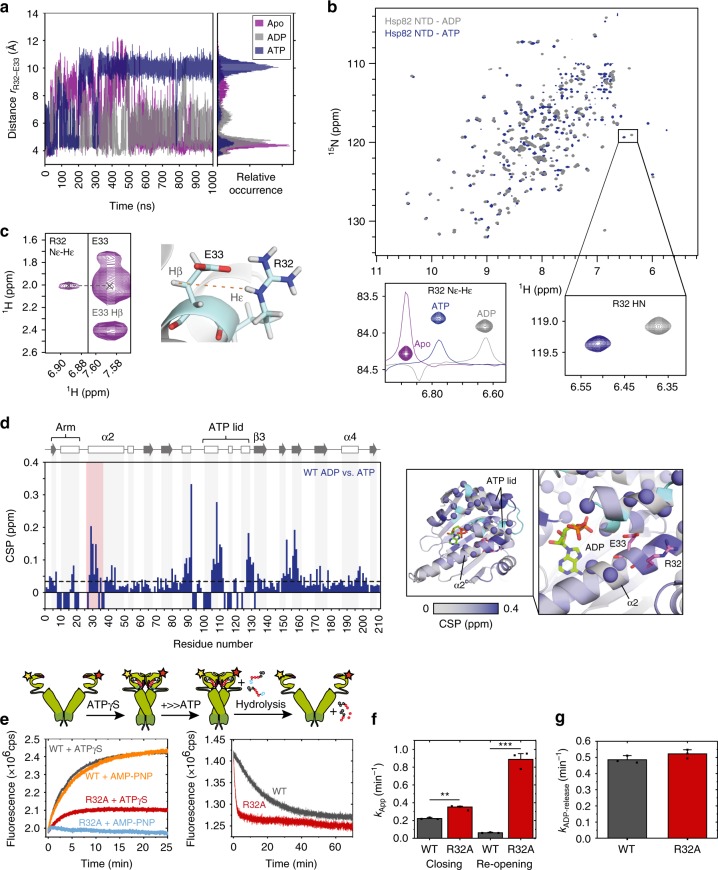
Biophysical characterization of Hsp90. **a** Arg-32/Glu-33 distance from classical atomistic MD simulations, showing that the ion pair is closed in the apo-form (purple) of the Hsp90-NTD and its conformation is sensitive to nucleotide binding (ADP in grey/ATP in blue). **b** ^1^H-^15^N-HSQC spectra of the Hsp90-NTD bound to ADP (grey) and ATP (blue), with close-ups of the resonances corresponding to the Arg-32 amide group (bottom right) and its sidechain Hε-Nε (bottom left), for which the ^1^H traces are also included (apo shown in purple). **c** NOE cross-peak between Arg-32 Hε and Glu-33 Hβ in the apo-NTD, confirming the spatial proximity of the sidechains. **d** Left: chemical shift perturbations (CSPs) of the Hsp90-NTD bound to ADP and ATP. Negative bars represent residues that could not be assigned due to conformational dynamics. Secondary structure elements are shown on the top and altered residues next to Arg-32 are highlighted in red. Right: distribution of CSP on the NTD for ATP/ADP exchange. Amides of residues that are not visible due to conformational dynamics are represented by blue spheres, whereas unassigned residues are shown in cyan. **e** FRET experiments show that closing (left) in Hsp90-R32A (in red and light blue) takes place to a smaller extent in comparison with the wild type (WT, in black and orange). Right: re-opening of the closed dimer takes place faster in Hsp90-R32A (red) than in WT Hsp90 (black). The schematic figure on the top illustrates the experimental procedure. **f** Rates for closing and re-opening of WT Hsp90 and Hsp90-R32A obtained from mono-exponential fitting of the FRET curves shown in **e**. Error bars represent SD from three independent measurements (*n* = 3), shown as black dots. Statistical significance was assessed using a two-sided two-sample *t*-test and a level of significance of 0.01 (**) and 0.001 (***). The fit yields a higher closure rate for R32A relative to WT Hsp90. **g** ADP-release assays show that WT Hsp90 and Hsp90-R32A have similar global ATP turnover. Error bars represent SD from three independent measurements (*n* = 3), shown as black dots. Source data are provided as a source data file.

To probe the conformational dynamics linked with the catalytic steps, we performed atomistic MD simulations for 250 ns of both the full-length Hsp90 dimer and a monomeric NM-domain model, as well as 1 μs MD simulations for models comprising only the NTD in different ligand states (Supplementary Figs. 5 and 6). Interestingly, we observe that Arg-32 undergoes a conformational change in the full-length Hsp90. In these simulations, Arg-32 flips away from Glu-33 and forms transient contacts with a network of charged/polar residues in the M-domain on 50–100 ns timescales (Fig. 3c and Supplementary Fig. 7). Opening of the Arg-32/Glu-33 ion pair increases the p*K*_a_ value of Glu-33 by ca. 10 p*K*_a_ units, indicating a strong influence of Arg-32 on the p*K*_a_ of Glu-33 (Supplementary Fig. 8). Although we do not expect to achieve exhaustive sampling of these transient states, the electrostatic models, nevertheless, also support that the proton affinity of Glu-33 strongly increases upon the ion-pair dissociation, consistently with the QM/MM results (Fig. 2a). In stark contrast to simulations of the full-length Hsp90, the Arg-32/Glu-33 ion pair remains closed during the MD simulations of our monomeric NM-domain model, built from a high-resolution NTD-dimerized crystal structure (see Methods), suggesting that the dimerization and global conformational state of Hsp90 affects the ion-pair dynamics. However, in simulations of the monomeric NTD, we find that replacing ADP by ATP triggers opening of the Arg-32/Glu-33 ion pair, mimicking the conformational dynamics of this region observed in the full-length Hsp90 (Fig. 4a). Analysis of the Arg-32/Glu-33 sidechain distance in a selection of Hsp90 crystal structures shows that this ion pair is closed in most of the structures, except in an ATP-bound structure of the NTD, supporting the results from our MD simulations (Supplementary Table 4). In simulations of the full-length Hsp90, we observe ion-pair opening only in one of the two Hsp90 protomers (Fig. 3c), but a systematic exploration of the phase space would be required to determine whether the domains truly operate asymmetrically^11^. However, to probe the reproducibility of our simulations, we performed 100 ns duplicate MD simulations for the NM-domain and the NTD models. These simulations support similar ion-pair dynamics as observed in the original simulations (Supplementary Fig. 9).

To probe how the ATP-hydrolysis reaction is affected by opening of the Arg-32/Glu-33 ion pair, we re-calculated the reaction energetics for this conformation. Although the ATP-hydrolysis reaction still takes place by a semi-concerted proton transfer and phosphate cleavage process in these models (Supplementary Fig. 10), the free-energy barrier is reduced to ca. 25 kcal mol^−1^ (Fig. 2a), consistent with electrostatic tuning of Glu-33. According to transition state theory, this barrier corresponds to a reaction rate in the hours timescales (see Methods). Moreover, substitution of the arginine by an alanine, R32A, mimicking a fully shielded and extended open ion-pair conformation, shows a free-energy barrier of ca. 18 kcal mol^−1^ (Fig. 2a), corresponding to a reaction rate in the seconds timescales according to transition state theory (*k* = 1.3 s^−1^, see Methods), and is thus comparable to experimentally observed turnover rates of Hsp90 in the ~0.1–1 min^−1^ timescales^10^. MD simulations of the full-length R32A suggest that the substitution leads to re-arrangement of ion pairs within the N–M interface (Supplementary Fig. 11). The R32A variant shows indeed more ion pairs between the two M-domains in comparison with the WT Hsp90, whereas less ion pairs and a weaker interaction energy is observed between the two NTDs of R32A in the MD simulations (Supplementary Fig. 12). Our findings thus suggest that the conformational changes associated with the contact between the NTD and M-domain strongly modulate the ATP-hydrolysis energetics (see below).

### Probing the conformational state of Arg-32

To gain insight into the conformational changes of Arg-32 in the NTD of Hsp90, we employed NMR spectroscopy in combination with MD simulations in different nucleotide-bound states. Based on the observation that replacing ADP by ATP may trigger opening of the Arg-32/Glu-33 ion pair (Fig. 4a), we measured ^1^H-^15^N heteronuclear single quantum coherence (HSQC) spectra of the NTD of yeast Hsp90 in the apo*-*form and bound to ADP and ATP. The Nε–Hε proton provides a good reporter for the sidechain of Arg-32, which appears in the apo-NTD as a single intense NMR signal, as expected for a sidechain with restricted mobility (Fig. 4b, bottom left panel). The presence of a Nuclear Overhauser effect (NOE) cross-peak between Arg-32 Hε and Glu-33 Hβ confirms the spatial proximity of the two sidechains in the apo-form (Fig. 4c), consistent with our MD simulations of the NTD in the apo state (Fig. 4a). Structural rigidity of the Arg-32 sidechain is supported by {^1^H}-^15^N steady-state heteronuclear NOE experiments, which are sensitive to fast (ns–ps) dynamics^30^. However, a relatively high value of 0.58 ± 0.04 indicates that the Arg-32 sidechain is not flexible, consistent with its interaction with Glu-33.

Interestingly, we find that nucleotide exchange induces significant chemical shift perturbations (CSPs) near the Arg-32 region (Fig. 4b, d) and in the ATP-lid, which is partially similar to previous results by Zhang et al.^31^ for human Hsp90, although the yeast protein studied here has several features that significantly differ from the human isoform. For example, in contrast to yeast Hsp90 (Fig. 4b, c), the neighbouring residues around the Arg-46 (Arg-32) region could not be assigned in the human Hsp90^31^, possibly due to signal broadening originating from dynamics on the micro- to millisecond timescales.

CSP around the Arg-32 residue could reflect a higher population of an open ion-pair conformation in the ATP-bound NTD, as predicted by the MD simulations (Fig. 4a). This notion is further supported by the sidechain heteronuclear NOE values, which decrease from 0.58 ± 0.09 for the ADP-bound NTD, to 0.48 ± 0.09 for the ATP-bound NTD. In addition, the increased line-width of the Hε signal observed in the ADP- and ATP-bound states (Fig. 4b) is consistent with dynamics at μs–ms timescales. Breaking the ion pair in the apo state of the NTD-R32A variant causes significant shifts not only in the environment of the mutation point but also at the C-terminal helix α4 and in the ATP lid, emphasizing its role on the structure and conformation of the NTD (Supplementary Fig. 13).

### Effects of the R32A mutation on Hsp90 conformation and function

To probe further how the computationally identified R32A substitution influences the global conformational structure and dynamics of Hsp90, we employed a FRET system with the NTDs labelled by fluorescent donor and acceptor dyes that induce resonance energy transfer upon closing of the Hsp90 dimer^6^. Although our current MD simulations and NMR data capture short- to medium-range conformational changes in the protein structure, FRET and SAXS provide powerful techniques to capture global effects linked with the R32A substitution, providing complementary data about the Hsp90 structure and function.

Our FRET experiments on the full-length Hsp90 show that the R32A substitution strongly inhibits the closing of the dimer (Fig. 4e). Interestingly, the non-hydrolysable ATP-analogue, adenylyl-imidodiphosphate (AMP-PNP), and the slowly hydrolysable ATPγS, induce a similar closing behaviour in the WT Hsp90, whereas the R32A variant forms only a partially closed FRET dimer with ATPγS. At the same time, re-opening of the R32A dimer takes place faster as compared with the WT Hsp90 (Fig. 4e). Although R32A-Hsp90 does not close to the same extent as the WT Hsp90 dimer, exponential fitting of the FRET data gives a higher closure rate for R32A-Hsp90 as compared with the WT (Fig. 4f). A possible explanation for this observation is that the partially closed R32A-Hsp90 dimer is formed faster than the fully closed WT Hsp90 dimer. This hypothesis is supported by a ca. 10-fold faster re-opening of the R32A-Hsp90 dimer relative to the WT Hsp90 dimer (Fig. 4e, f). The ratio of the apparent rates (Supplementary Table 5) is consistent with a lower population of the closed conformation in R32A and thus indirectly supports a similar or even lowered intrinsic ATP-hydrolysis barrier in the R32A variant relative to WT Hsp90.

**Fig. 5:**
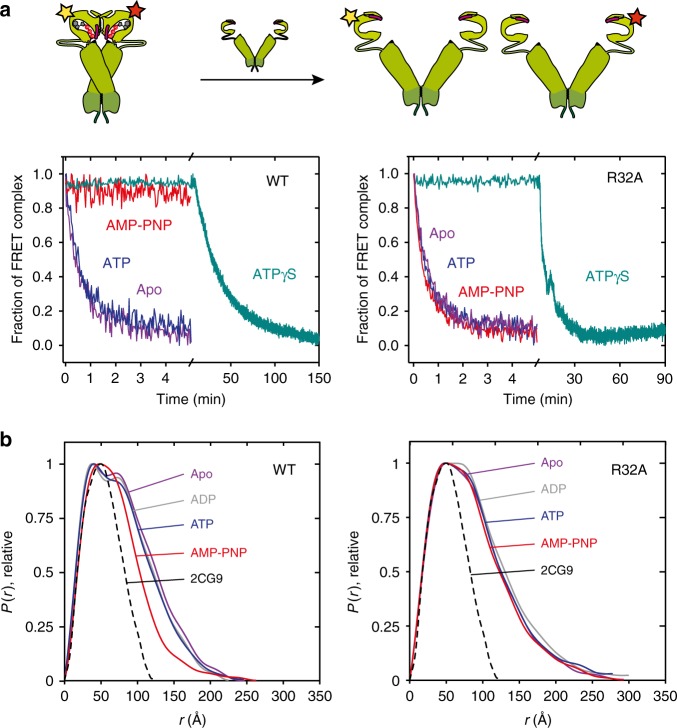
Characterization of the Hsp90 dimer by FRET and SAXS experiments. **a** Chase experiments with the closed wild type (WT, left) and R32A (right) Hsp90 dimers in the apo-form and bound to different nucleotides (ATP, AMP-PNP, ATPγS). Chase experiments were initiated by addition of unlabelled Hsp90. **b** Left: pair distance distributions (*P*(*r*)) obtained from the SAXS profiles of apo wild-type (WT) Hsp90 (purple) and bound to different nucleotides (ADP, ATP, AMP-PNP). The *P*(*r*) distribution calculated from the X-ray structure of Hsp90 in the closed conformation (PDB ID: 2CG9)^9^ is shown for comparison (in black). Right: pair distance distributions of the R32A variant in different nucleotide states. AMP-PNP favours a compact conformation of wild-type (WT) Hsp90, whereas the R32A variant shows no major conformational rearrangements.

In chase experiments (Fig. 5a), where we used unlabelled yeast Hsp90 (Hsp82) to disrupt the preformed FRET dimer^6^, the WT Hsp90 shows no subunit exchange from its closed compact conformation in the presence of AMP-PNP, in line with previous observations^6,13^. In stark contrast, the R32A variant has a significant subunit exchange, consistent with the perturbed closing equilibrium of the mutant. These findings suggest that the closed R32A dimer is not stabilized as strongly as the closed dimer of WT Hsp90. The perturbed closing also affects the co-chaperone binding of Sba1, which is important for the fine-tuning of the chaperone cycle and stabilizes the closed conformation (Supplementary Fig. 14)^32^. To this end, Sba1 shows a lower binding affinity for the R32A variant as compared with the WT Hsp90.

To obtain further structural information of the R32A variant, we performed in-line size-exclusion chromatography coupled to SAXS experiments. The analysis of the scattering profiles with the Guinier approximation and the pair-wise distance distribution functions (*P*(*r*)) shows that the apo-form of both the WT and R32A variant of the full-length Hsp90, populate an ensemble of a fully open conformation with a radius of gyration (*R*_g_) of ca. 62–66 Å (Fig. 5b, Supplementary Fig. 15, and Supplementary Tables 6 and 7). Nucleotide binding to the WT enzyme induces an overall decrease in the *R*_g_ to 59.4 Å and 57.6 Å for the ADP- and ATP-bound states, respectively, leading to a more compact conformation, consistent with previous observations^33^. However, in stark contrast to the WT enzyme, ligand binding triggers no significant structural changes in the R32A variant, suggesting that the coupling between the NTD and M-domain is drastically perturbed, thus affecting the overall conformational state of the Hsp90 dimer. The involvement of Arg-32 in the closing process is particularly pronounced upon binding of the non-hydrolysable AMP-PNP, which induces a compact Hsp90 conformation in the WT enzyme (*R*_g_ = 53.1 Å), reducing the inter-atomic distance distribution, but leaves the global conformation of R32A largely unaffected (*R*_g_ = 62.3 Å; Fig. 5b and Supplementary Tables 6 and 7). These observations support the notion that the free energy from the nucleotide binding site is not effectively transduced into the conformational state in the R32A variant.

To probe the influence of the R32A substitution in vivo, we used a plasmid shuffling approach to introduce the Hsp90 variant as the sole source of Hsp90 in the yeast *Saccharomyces cerevisiae*, where Hsp90 is essential for cell survival. Interestingly, the shuffling experiments show that the R32A strain is not viable (Supplementary Fig. 16), possibly due to the impaired closing dynamics, which is crucial for the maturation and regulation of client proteins, suggesting that the R32A variant cannot fulfil its tasks in the cell. A possible explanation for this observation is that R32A does not adopt the fully closed conformation, which is necessary for the activation of client proteins^7,9^.

As our calculations predict a lower catalytic barrier for the ATP-hydrolysis in R32A-Hsp90, we tested the interaction of R32A-Hsp90 with ATP. The R32A variant binds ATP to a similar extent as the WT Hsp90 (Supplementary Fig. 17) and is able to hydrolyse ATP, consistent with its lowered catalytic barrier (Fig. 2a), but remarkably, the ADP-release kinetics in the R32A variant (0.55 ± 0.05 min^−1^) is practically identical to that of the WT Hsp90 (0.50 ± 0.05 min^−1^) (Fig. 4g). The NM-fragment of R32A-Hsp90 does not, however, show ATPase activity, which is also lacking from the NM construct of the WT protein (Supplementary Fig. 18). Moreover, the coupling between the NTD and the M-domain in the full-length Hsp90 is indirectly supported by our experiments using the E381Q variant, which show an increased closing rate and ATPase activity relative to the WT enzyme (Supplementary Fig. 19). We speculate that the E381Q substitution could, however, also affect the conformation of R380, which has been linked to formation of the “closed 2” state^13,29^.

Interestingly, despite the perturbed closing dynamics, we observe that the R32A variant shows similar ADP-release kinetics as the WT Hsp90. To find an explanation for the seemingly contradicting experimental results, we constructed a simplified kinetic model of the Hsp90 cycle (Supplementary Fig. 20 and Supplementary Table 8). Although the intrinsic ATPase rate is expected to increase as a result of the R32A substitution, our kinetic models suggest that the overall ADP-release rate could remain unchanged if the population of the closed conformation is reduced at the same time, as also suggested by our FRET data (Fig. 4e, f). The kinetic models suggest that even a 100-fold rate increase in the catalytic step, would require only a two- to threefold reduction in formation of the fully closed conformation to account for the observed ADP-release data. In other words, although the intrinsic ATPase reaction is strongly modulated by the conformational state of Hsp90, the less efficient population of the closed conformation could account for the unchanged ADP-release rate. The R32A variant is, however, likely to undergo a more complex reaction cycle than that modelled here, but elementary rates for the transitions remain unknown. Nevertheless, taken together, our combined findings suggest that the Arg-32 site has drastic effects on both the local and global conformational dynamics of Hsp90, which in turn is directly coupled to the reaction barrier for the ATP-hydrolysis process.

## Discussion

We have shown here that the catalysis leading to the hydrolysis of a phospho-anhydride bond within the active site of Hsp90 is modulated by both local and global conformational changes in the protein. Based on molecular simulations, we identified that Arg-32 within the active site functions as a key coupling element that mediates the communication across the different domains of Hsp90. The ATPase reaction barrier is electrostatically tuned by conformational transitions in Arg-32 and, due to microscopic reversibility, perturbation of the catalytic machinery also results in an impaired global closing behaviour that is central for the chaperone cycle. Our data indicate that the conformational switching takes place via a conserved network of ion pairs between the N- and M-domains of the enzyme involving, e.g., E381 (Supplementary Figs. 7 and 19), with mechanistic similarities to, e.g., respiratory complex I, a mitochondrial redox-driven proton pump where quinone reduction triggers proton pumping, up to 200 Å from the active site, by conformational changes in a network of conserved ion pairs^34–37^.

It is puzzling that although the R32A variant is not viable in vivo and unable to properly form a closed compact state, the enzyme still hydrolyses ATP with an unchanged ADP-release rate. The catalytic reaction is not considered rate-limiting for the WT Hsp90 machinery, but is normally limited by the closing kinetics^6,38^. However, our calculations showed that the ATPase reaction has a significant barrier also in the WT Hsp90, especially prior to conformational changes within Arg-32 that are captured by both our MD simulations and NMR experiments. In contrast to the WT enzyme, our R32A variant has a significantly perturbed closing equilibrium, but it is still able to hydrolyse ATP with an overall unchanged cycle turnover. These findings indicate that Arg-32 functions as a switching point, the perturbation of which decouples the catalysis from global conformational changes. To this end, our kinetic models support that the ADP-release rate could indeed remain unchanged if the closing kinetics is perturbed at the same time (Supplementary Fig. 20), as also supported by our FRET experiments (Fig. 4e, f).

**Fig. 6:**
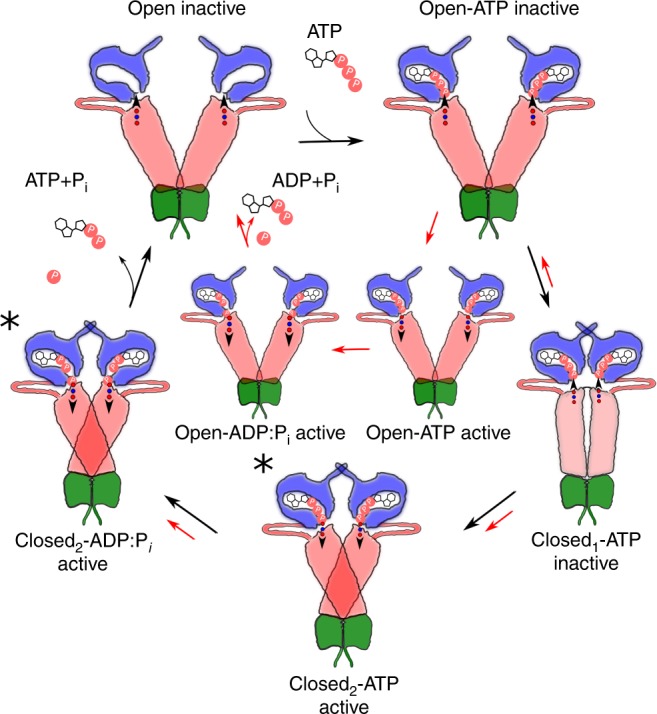
Putative Hsp90 reaction cycle. ATP-binding to the open, catalytically inactive state, induces formation of a compact ATP-bound state (closed_1_-ATP inactive). Conformational changes (red/blue dots followed by arrow) within the NTD and M-domain lead to a catalytically active state (active) that triggers formation of a fully compact active form of Hsp90 (closed_2_-ATP active), with chaperone activity (marked with an asterisk). The hydrolysis reaction leads to splitting of the phospho-anhydride bond and formation of the closed_2_-${{\mathrm{ADP}} \hskip -2.5pt : \hskip -2.5pt{\mathrm{P}}_{\mathrm{i}}}$ state, which further dissociates to the open state. The R32A decoupling variant lowers the barrier for switching between inactive and active catalytic states that also lowers the ATP-hydrolysis barrier, but increases the free energy of forming the compact state (red arrows). The decoupling mutant could also run an ATP-hydrolysis cycle in parallel to the regular cycle that is fully decoupled from the formation of the closed form (red arrows). The NTD is shown in blue, the middle domain in red, and the CTD in green.

The R32A variant could, however, also run another, fully decoupled cycle in parallel to the regular Hsp90 cycle, where the ATP-hydrolysis reaction is independent of the formation of a closed compact state (Fig. 6). Binding of the co-chaperone Sba1 to R32A supports that the closed conformation is populated at least to some extent in the mutant (Supplementary Fig. 14) and kinetic competition between these cycles is thus consistent with the non-viable, but catalytically active R32A variant. This suggests that R32A-Hsp90 could hydrolyse ATP in the (partly) open state, suggesting that this variant might also have an increased ATPase activity in an NM-fragment. We were, however, unable to detect ATP catalysis in the NM-fragment in either the WT or R32A variant (Supplementary Fig. 18), suggesting that the NM construct might sample different conformations than the full-length Hsp90 dimer, as dimerization initiated at the CTD is most likely required for correctly organizing the active site.

In conclusion, by using an integrated computational and experimental approach, we showed here how the catalytic activity couples to conformational changes in the molecular chaperone Hsp90. We identified a central ion pair that switches between the domain harbouring the active site and the M-domain of the enzyme, mediating long-range signals across the protein framework. Conformational changes in this ion pair favour the ATP-hydrolysis reaction by electrostatic tuning. The computationally derived R32A variant was experimentally expressed and its properties were probed by NMR, SAXS, and FRET experiments. The R32A mutation impedes the formation of a compact Hsp90 dimer conformation that, in turn, is a pre-requisite for the chaperone activity. Substitution of the identified site decouples catalysis from global conformational changes, leading to a non-viable mutant that hydrolyses ATP without activating the chaperone cycle as indicated by our in vivo experiments. These results open up possibilities to unravel the exact molecular principles on how the energy from the ATPase/closed state is transduced into the chaperone activity in Hsp90. Our combined data thus highlight new features of the remarkable coupling between catalysis and biological activity in Hsp90.

## Methods

### DFT and QM/MM models of the active site in Hsp90

Monomeric QM and QM/MM models were constructed based on the crystal structure of Hsp90 from *Danio rerio* (PDB ID: 4IVG)^39^. The calculations were performed at the B3LYP-D3/CHARMM36 (QM/MM) and B3LYP-D3 (QM) level using def2-SVP/def2-TZVP (Mg) basis sets^40–43^. The QM region comprised ATP, Mg^2+^, Arg-32, Glu-33, Asn-37, Ser-99, and Arg-380, as well as six water molecules, in addition to the backbone of residues 118–124 that was included only in the QM models (see Supplementary Table 9 for residue numbering in different species). Link atoms were introduced between the Cβ and Cα atoms in the QM/MM models, whereas terminal carbon atoms were kept fixed during structure optimization in the QM models. In the QM models, the protein surroundings were treated as a polarizable medium with a dielectric constant of *ε* = 4^44^ and reaction pathways were optimized using a chain-of-state method^45^. Vibrational and entropic contributions were estimated at the B3LYP-D3/def2-SVP/def2-TZVP (Mg)/*ε* = 4 level by calculating the Hessian numerically^46^. The catalytic tuning effect of Arg-32 was studied by scanning the distance between the Cζ of Arg-32 and Cδ of Glu-33 (see Supplementary Fig. 4). Single-point calculations with def2-TZVP basis set were performed on the reactant, transition state, and product structures extracted from the reaction path optimizations. The QM/MM system with ca. 17,000 atoms was cut out from the full protein, using a sphere with a 30 Å radius around the ATP site, and embedded in a water sphere with 150 mM NaCl. Reaction pathways were optimized with the MM region fixed. Energy decomposition was carried out by removing individual residues from the QM/MM system during single-point calculations, without re-optimizing the protein structure. Based on the reaction profiles, 27 ps restrained QM/MM MD simulations were performed with harmonic restraints on the commonly employed reaction coordinate describing the relevant bond-formation and bond-breaking processes^27,28^, *R* = *r*_4_ – *r*_3_ + *r*_2_ – *r*_1_ = [−2.9 Å, 2.6 Å] (see Fig. 2b), using force constants of 100 or 500 kcal mol^−1^ Å^−2^. All atoms beyond 15 Å from the QM region were kept fixed in the umbrella sampling (US) simulations. The statistical overlap of the sampled reaction coordinate is shown in Supplementary Fig. 1. Free-energy profiles were computed using the US/weighted histogram analysis method (WHAM) with a convergence criterion of 0.00001 kcal mol^−1^^47^. Bootstrap error analysis was performed within WHAM with ten Monte Carlo (MC) trial steps to estimate the statistical uncertainty of the free energy. Rate constants were calculated using the Eyring equation at *T* = 310 K using a standard pre-exponential factor,

1 $$k = \kappa \frac{{k_BT}}{h}e^{ - \frac{{\Delta G \ast }}{{RT}}}$$

where *κ* is the reflection coefficient that was set equal to 1, ΔG* is the free-energy barrier, *R* is the gas constant, *k*_B_ is Boltzmann’s constant, and *h* is Planck’s constant. All DFT calculations were performed using TURBOMOLE^48^, which was coupled together with CHARMM in the QM/MM models^49,50^.

### Classical MD simulations of Hsp90

Classical MD simulations were performed on the full-length dimeric Hsp90 model and the R32A mutant constructed based on the dimeric crystal structure of yeast Hsp90 (PDB ID: 2CG9)^9^. MD simulations were also carried out for the monomeric Hsp90 model with NM-domains constructed based on the X-ray structure of the dimeric NM-fragment of Hsp90 from *D. rerio* (PDB ID: 4IVG)^39^ and NTD models of yeast Hsp90 in the apo state and with ADP or ATP (PDB ID: 1AMW)^51^. The protein was embedded in a water-ion environment with 100–150 mM NaCl. The complete simulation setups comprised ca. 72,300 atoms (NM-model), 77,300 atoms (NTD model), and 303,000 atoms (full-length dimer). The constructs were simulated for 250–1000 ns at *T* = 310 K, using a 2 fs timestep and keeping all covalently bound hydrogens fixed. We employed the CHARMM27/36 force field^43,52^ treating the long-range electrostatics by the Particle Mesh Ewald approach. The MD simulations were performed using NAMD^53^ and Visual Molecular Dynamics (VMD)^54^ was used for visualization and analysis. All Arg-32/Glu-33 distances shown in the paper are measured between the Cζ of Arg-32 and the Cδ of Glu-33. All simulations are summarized in Supplementary Table 10.

### Calculation of p*K*_a_ values

Poisson-Boltzmann (PB) continuum electrostatic calculations with MC sampling of 2^*N*^ protonation states of the full-length Hsp90 dimer were used for the calculation of p*K*_a_ values. The Adaptive Poisson-Boltzmann Solver (APBS) was used for the PB calculations^55^ and Karlsberg+ was employed for the MC sampling^56,57^. The protein was described with atomic partial charges, embedded in an inhomogeneous dielectric continuum with a dielectric constant of *ε* = 4. Bulk water was approximated by a homogeneous dielectric continuum with *ε* = 80. The molecular surface routine in APBS was used to calculate the boundary interface between the protein and the solvent, using a solvent probe radius of 1.4 Å and modelling an implicit ionic strength of the solvent of 100 mM KCl. Protonation probabilities were calculated every ns along the 250 ns MD simulation of the full-length Hsp90 dimer.

### Kinetic modelling

Kinetic models of a simplified chaperone cycle of the WT and R32A variant were simulated using the stochastic Gillespie algorithm in Dizzy^58^. Kinetic parameters are given in Supplementary Table 8.

### Protein purification

The R32A, E381Q, and D61C (for FRET) single point mutations were introduced by site-directed mutagenesis according to the NEBase Changer protocol in pET28 vectors (Invitrogen, Karlsruhe, Germany) containing the yeast Hsp90 (Hsp82) sequence. Hsp90-NM constructs (aa 1–529) were cloned using a pET28a-SUMO Vector containing an N‐terminal 6×His-SUMO-tag. For the NTD constructs (aa 1–210), a pETM-11 vector was used. The plasmids were transformed into the *Escherichia coli* strain BL21 (DE3) cod+ (Stratagene, La Jolla, USA). Full-length proteins (yeast Hsp90, sba1) were expressed for 4 h at 37 °C, NM constructs were expressed at 25 °C overnight. Expression was induced with 1 mM Isopropyl β-d-1-thiogalactopyranoside. After collection, the cells were re-suspended in Ni-NTA buffer A (50 mM NaH_2_PO_4_, 500 mM NaCl, 10 mM imidazole pH 7.5) supplemented with EDTA-free protease inhibitor [SERVA] and DNase1. Cells were lysed using a Cell Disruption System (Constant Systems) at 1.8 kbar. After lysate clarification, the supernatant was loaded on a 5 mL HisTrap HP column (GE Healthcare) and washed with 10 CV Ni-NTA buffer A and 10 CV 5 % Ni-NTA buffer B (50 mM NaH_2_PO_4_, 500 mM NaCl, 300 mM imidazole pH 7.5). The bound proteins were eluted with 100 % Ni-NTA buffer B. Full-length proteins were pooled, diluted to 150 mL with ResQ buffer A (40 mM HEPES, 20 mM KCl, 1 mM EDTA, 1 mM dithiothreitol (DTT) pH 7.5) and loaded onto a ResQ column. NM constructs were supplemented with His_6_-tagged SUMO-protease after Ni-NTA and dialysed against 5 L Ni-NTA buffer A ON at 4 °C. The protein solution was again loaded on a 5 mL HisTrap HP column and the flowthrough was collected. As a final step, proteins were loaded on a Superdex 16/60 75 pg SEC column (GE Healthcare) and eluted with SEC buffer (40 mM HEPES, 150 mM KCl, 5 mM MgCl_2_ pH 7.5). For the NMR samples (NTD constructs), cells were grown in M9 minimal media supplemented with ^15^NH_4_Cl and ^13^C_6_-glucose (Aldrich, Germany), with protein expression and purification conducted following the procedure for the NM constructs. Instead of SUMO-protease, TEV-protease was used. Buffers used were buffer A (50 mM Tris-HCl pH 8, 150 mM NaCl, 5 mM imidazole and 0.02 % NaN_3_), buffer B (50 mM Tris-HCl pH 8, 150 mM NaCl, 500 mM imidazole and 0.02 % NaN_3_), and dialysis buffer (20 mM Tris-HCl pH 8, 300 mM NaCl, 2 mM DTT). For SEC, NMR buffer (see “NMR experiments”) was used. The primer sequences are listed in Supplementary Table 11.

### ADP-release assays

The ATPase activity was measured spectrophotometrically by following the ADP-release reaction using an enzymatic ATP regenerating system^59^. Full-length (3 μM) yeast Hsp90 (Hsp82) and 10 μM of the NM constructs were used in 40 mM HEPES (pH 7.5), 150 mM KCl, 5 mM MgCl_2_. Measurements were performed in a Cary 100 UV-Vis photometer (Varian, Inc.) at 30 °C. The reaction was initiated by the addition of 2 mM (full-length Hsp90) and 4 mM (Hsp90-NM) ATP. For the determination of *K*_M_-values (ATP-binding), different concentrations of ATP from 0.1 mM to 5 mM were used. To subtract the background activity, 50 μM radicicol were added at the end of the measurement. The data were analysed by linear regression using Origin 8.0. ADP-release kinetics were determined using the following equation, where *m* is the slope, *ε*_NADH_ is the extinction coefficient of NADH, and *c*_Hsp90_ is the yeast Hsp90 (Hsp82) concentration:

2 $$k_{\mathrm{{ADP - release}}} = \frac{{ - m}}{{({\upvarepsilon}_{{\mathrm{NADH}}} \cdot c_{{\mathrm{Hsp}}90})}}$$

For the determination of *K*_M_-values for ATP binding, the ADP-release kinetics were plotted against the respective ATP concentration. The data were fitted using the Michaelis–Menten equation:

3 $$y = \frac{{V{\mathrm{{max}}} \cdot x}}{{K_{\mathrm{{M}}} + x}}$$

### FRET experiments

FRET experiments were conducted following the protocol of Hessling et al.^6^. Atto488 (donor) and Atto550 (acceptor) (ATTO-TEC GmbH) labelled yeast Hsp90-D61C (200 nM) was used. Measurements were performed in 40 mM HEPES (pH 7.5), 150 mM KCl, 5 mM MgCl_2,_ 2 mM DTT in a Fluoromax 4 fluorescence spectrophotometer (Horiba Jobin Yvon) at 30 °C. Closing of Hsp90 was induced by addition of 2 mM AMP-PNP or 2 mM ATPγS. Chase experiments were performed with a tenfold excess (4 µM) of unlabelled WT yeast Hsp90 (Hsp82) to disrupt the FRET complex. Samples were incubated for 60 min at 30 °C prior to the addition of the unlabelled species. For determination of re-opening rates, closing was induced with 2 mM ATPγS. After the equilibrium was reached, a tenfold excess (20 mM) of ATP was added, to induce re-opening of the Hsp82 dimer. The data were analysed with Origin 8.0 and fitted using a mono-exponential equation:

4 $$y = A_{1}^{(\frac{{ - x_{1}}}{{t_{1}}})} + y_{0}$$

5 $$k_{\mathrm{{App}}} = \frac{1}{{t_{1}}}$$

### Yeast viability assay of Hsp82 variants

Plasmid shuffling experiments were conducted following the protocol of Nathan et al.^60^, using the ∆PCLDα *S. cerevisiae* strain from S. Lindquist’s laboratory deficient in genomic Hsp82 and Hsc82 containing a plasmid coding for WT Hsp82. The pKAT6 plasmid is constitutively expressed under the control of the glycerinaldehyde-3-phosphate dehydrogenase gene promotor (GPD promotor) and carries a URA selection marker for the selection of cells that have lost the WT Hsp82 plasmid in the medium supplemented with 5-Fluoroorotic Acid (5-FOA) (Thermo Fisher Scientific). The cells were transformed with either the empty vector p413 (negative control), the p413 vector, coding for the WT Hsp82 (positive control), and the p413 vector, coding for the Hsp82 R32A variant. Hsp82 is essential for yeast survival and the loss of the pKAT6 plasmid, due to 5-FOA-induced selection, inhibits yeast growth. Transformation of p413 vectors containing Hsp82 variants might restore yeast growth, depending on the characteristics of the Hsp82 variants.

### Fluorescence anisotropy

Fluorescence anisotropy experiments were used to probe the binding of sba1 to WT yeast Hsp90 and the R32A variant. Measurements were conducted in a JASCO-8500 fluorescence spectrophotometer with polarisers (Jasco, Groß-Umstadt, Germany) at 30 °C in 40 mM HEPES (pH 7.5), 150 mM KCl, 5 mM MgCl_2_, containing 2 mM AMP-PNP. Excitation and emission wavelengths were set to 490 and 530 nm, respectively, using 200 nM of Atto488-labelled sba1 and 500 nM Hsp90. After pre-incubation of Hsp90 with labelled sba1, unlabelled sba1 was added in varying concentrations to compete out the preformed labelled sba1–Hsp90 complex. The *K*_D_-value was determined by fitting the anisotropy signal, *r*, to the sba1 concentration, *c*_sba1_,

6 $$r = F_P - (c_{{\mathrm{Hsp}}90} + c_{{\mathrm{sba}}1} + K_D) - \left( {\frac{{F_{\mathrm{P}} - F_{{\mathrm{PL}}}}}{{2c_{{\mathrm{Hsp}}90}}}} \right)\sqrt {(c_{{\mathrm{Hsp}}90} + c_{{\mathrm{sba}}1} + K_D)^2 - 4c_{{\mathrm{Hsp}}90}c_{{\mathrm{sba}}1}}$$

where *F*_P_ is the anisotropy of unbound labelled sba1, *F*_PL_ is the anisotropy of the labelled sba1–Hsp90 complex, and *c*_Hsp90_ is the concentration of yeast Hsp90.

### SAXS experiments

SAXS data were collected at beamline BM29 at the European Synchrotron Radiation Facility (Grenoble, France). Fifty microlitres of sample in 25 mM Hepes pH 7.5, 150 mM KCl, 5 mM MgCl_2_, 1 mM TCEP, 0.02 % NaN_3_ was injected to a Superdex 200 5/100 GL column (GE Healthcare) connected online to the SAXS capillary. One SAXS frame per second was recorded at a flow rate of 0.15 ml min^−1^. Nucleotide-bound forms were prepared by adding the compounds at a final concentration of 2.5 mM followed by incubation at room temperature. Due to the fast *k*_off_ of the nucleotides, the buffers employed in the chromatographic runs contained 2.5 mM ADP, 2.5 mM ATP, and 1 mM AMP-PNP. SEC-SAXS chromatograms were analysed using the Chromixs software^61^. Briefly, >100 buffer frames with constant average intensity were selected. Sample frames were selected from the chromatogram based on (1) chromatographic peak shape and (2) constant *R*_g_ across the selected region. The subtracted averaged scattering profiles were analysed using the Primus software package^62^ to extract the *R*_g_ values and the *P*(*r*) distributions were obtained using the Gnom programme^63^. Theoretical scattering profiles were computed from X-ray coordinates using Crysol^64^.

### NMR experiments

NMR spectra were recorded using Bruker 500, 600, and 950 MHz spectrometer (Bruker, Billerica, USA) at 25 °C, using an NMR buffer with 20 mM sodium phosphate, 100 mM NaCl, 5 mM MgCl_2_, 5% D_2_O, and 0.2% NaN_3_ pH 6.5, and a protein concentration between 500 and 600 μM. ADP was added at a final concentration of 5 mM, whereas ATP was added at 2.5 mM together with an ATP regenerating system^65^. These saturating conditions were used to avoid interference of different nucleotide affinities in the WT and mutant constructs. Chemical shift assignments were based on previous work for the Hsp90-NTD^33,66^ and were extended for the R32A mutant and the nucleotide-bound forms using a combination of triple-resonance HNCA, HNCOCA, and HNCACB experiments using non-uniform sampling^67^. Spectra were processed using NMRPipe^68^ and analysed using the CCPnmr software^69^. CSPs were calculated for backbone amide peaks of 2D ^1^H,^15^N-HSQC correlation experiments using the equation,

7 $$\Delta \delta _{\mathrm{{N,H}}}\left( {\mathrm{{ppm}}} \right) = \sqrt {\Delta \delta _{\mathrm{{H}}}^2 + \left( {\alpha \cdot \Delta \delta _{\mathrm{{N}}}} \right)^2}$$

where *α* is a scaling factor, calculated from the ratio between the ^1^H and ^15^N chemical shift ranges (*α* = 0.1689). To observe the arginine guanidine sidechain NMR signals in ^1^H-^15^N-HSQC spectra, the ^15^N carrier frequency was centred at 105 p.p.m. with a spectral width set to 70 p.p.m. {^1^H}-^15^N steady-state heteronuclear NOE experiments were performed using modified sequences described by Farrow et al.^30^ by collecting two datasets, with and without ^1^H saturation, respectively. Heteronuclear NOE values were obtained from the intensity ratio of R32 Hε–Nε peaks between the saturated and unsaturated spectra, and errors were estimated from spectral baseplane noise root-mean-square deviation (RMSD) as described by Farrow et al.^30^. ^15^N-edited NOESY experiments were performed using standard sequences with a mixing time of 120 ms^70^. The NMR assignments of the NTD of the R32A variant have been deposited in the BMRB under the accession number 27858.

### Reporting summary

Further information on research design is available in the Nature Research Reporting Summary linked to this article.

## Supplementary information

Supplementary Information

Reporting Summary

## Data Availability

The NMR assignments for the N-terminal domain of yeast Hsp90-R32A have been deposited in the BMRB under the accession number 27858. The source data underlying Fig. 4e-g and Supplementary Figs. 14, 17, 18, and 19 are provided as a Source Data file. Other data are available from the corresponding author upon reasonable request.

## Source Data

Source Data

## References

[1] Nathan DF, Vos MH, Lindquist S (1997). *In vivo* functions of the *Saccharomyces cerevisiae* Hsp90 chaperone. Proc. Natl. Acad. Sci. USA.

[2] Picard D (2002). Heat-shock protein 90, a chaperone for folding and regulation. Cell. Mol. Life Sci..

[3] McClellan AJ (2007). Diverse cellular functions of the Hsp90 molecular chaperone uncovered using systems approaches. Cell.

[4] Meyer P (2003). Structural and functional analysis of the middle segment of Hsp90: implications for ATP hydrolysis and client protein and cochaperone interactions. Mol. Cell.

[5] Wegele H, Muschler P, Bunck M, Reinstein J, Buchner J (2003). Dissection of the contribution of individual domains to the ATPase mechanism of Hsp90. J. Biol. Chem..

[6] Hessling M, Richter K, Buchner J (2009). Dissection of the ATP-induced conformational cycle of the molecular chaperone Hsp90. Nat. Struct. Mol. Biol..

[7] Southworth DR, Agard DA (2011). Client-loading conformation of the Hsp90 molecular chaperone revealed in the Cryo-EM structure of the human Hsp90:Hop complex. Mol. Cell.

[8] Verba KA (2016). Atomic structure of Hsp90-Cdc37-Cdk4 reveals that Hsp90 traps and stabilizes an unfolded kinase. Science.

[9] Ali MMU (2006). Crystal structure of an Hsp90-nucleotide-p23/Sba1 closed chaperone complex. Nature.

[10] Panaretou B (1998). ATP binding and hydrolysis are essential to the function of the Hsp90 molecular chaperone *in vivo*. EMBO J..

[11] Elnatan D (2017). Symmetry broken and rebroken during the ATP hydrolysis cycle of the mitochondrial Hsp90 TRAP1. eLife.

[12] Grenert JP, Johnson BD, Toft DO (1999). The importance of ATP binding and hydrolysis by Hsp90 in formation and function of protein heterocomplexes. J. Biol. Chem..

[13] Zierer BK (2016). Importance of cycle timing for the function of the molecular chaperone Hsp90. Nat. Struct. Mol. Biol..

[14] Jackson AP, Maxwell A (1993). Identifying the catalytic residue of the ATPase reaction of DNA gyrase. Proc. Natl. Acad. Sci. USA.

[15] Okimoto N (2001). Theoretical studies of the ATP hydrolysis mechanism of myosin. Biophys. J..

[16] Hayashi S (2012). Molecular mechanism of ATP hydrolysis in F_1_-ATPase revealed by molecular simulations and single-molecule observations. J. Am. Chem. Soc..

[17] Picard D (1990). Reduced levels of hsp90 compromise steroid receptor action *in vivo*. Nature.

[18] Colombo G, Morra G, Meli M, Verkhivker G (2008). Understanding ligand-based modulation of the Hsp90 molecular chaperone dynamics at atomic resolution. Proc. Natl. Acad. Sci. USA.

[19] Cunningham CN, Krukenberg KA, Agard DA (2008). Intra- and intermonomer interactions are required to synergistically facilitate ATP hydrolysis in Hsp90. J. Biol. Chem..

[20] Mickler M, Hessling M, Ratzke C, Buchner J, Hugel T (2009). The large conformational changes of Hsp90 are only weakly coupled to ATP hydrolysis. Nat. Struct. Mol. Biol..

[21] Taipale M (2012). Quantitative analysis of HSP90-client interactions reveals principles of substrate recognition. Cell.

[22] Mollapour M (2014). Asymmetric Hsp90 N domain SUMOylation recruits Aha1 and ATP-competitive inhibitors. Mol. Cell.

[23] Kamerlin SCL, Florián J, Warshel A (2008). Associative versus dissociative mechanisms of phosphate monoester hydrolysis: on the interpretation of activation entropies. ChemPhysChem.

[24] Abrahams JP, Leslie AGW, Lutter R, Walker JE (1994). Structure at 2.8 Å resolution of F_1_-ATPase from bovine heart mitochondria. Nature.

[25] Ban C, Yang W (1998). Crystal structure and ATPase activity of MutL: implications for DNA repair and mutagenesis. Cell.

[26] Schmidt H, Carter AP (2016). Structure and mechanism of the dynein motor ATPase. Biopolymers.

[27] Cheng Y, Zhang Y, McCammon JA (2005). How does the cAMP-dependent protein kinase catalyze the phosphorylation reaction: an ab initio QM/MM study. J. Am. Chem. Soc..

[28] Lopata A (2015). Mutations decouple proton transfer from phosphate cleavage in the dUTPase catalytic reaction. ACS Catal..

[29] Cunningham CN, Southworth DR, Krukenberg KA, Agard DA (2012). The conserved arginine 380 of Hsp90 is not a catalytic residue, but stabilizes the closed conformation required for ATP hydrolysis. Protein Sci..

[30] Farrow NA (1994). Backbone dynamics of a free and a phosphopeptide-complexed Src homology 2 domain studied by ^15^N NMR relaxation. Biochemistry.

[31] Zhang H (2015). A dynamic view of ATP-coupled functioning cycle of Hsp90 N-terminal domain. Sci. Rep..

[32] Schopf FH, Biebl MM, Buchner J (2017). The HSP90 chaperone machinery. Nat. Rev. Mol. Cell Biol..

[33] Lorenz OR (2014). Modulation of the Hsp90 chaperone cycle by a stringent client protein. Mol. Cell.

[34] Sharma V (2015). Redox-induced activation of the proton pump in the respiratory complex I. Proc. Natl. Acad. Sci. USA.

[35] Di Luca, A., Gamiz-Hernandez, A. P. & Kaila, V. R. I. Symmetry-related proton transfer pathways in respiratory complex I. *Proc. Natl. Acad. Sci. USA* **114**, E6314–E6321 (2017). 10.1073/pnas.1706278114 PMC5547640 28716925

[36] Warnau J (2018). Redox-coupled quinone dynamics in the respiratory complex I. Proc. Natl. Acad. Sci. USA.

[37] Kaila VRI (2018). Long-range proton-coupled electron transfer in biological energy conversion: towards mechanistic understanding of respiratory complex I. J. R. Soc. Interface.

[38] Richter K (2008). Conserved conformational changes in the ATPase cycle of human Hsp90. J. Biol. Chem..

[39] Lavery LA (2014). Structural asymmetry in the closed state of mitochondrial Hsp90 (TRAP1) supports a two-step ATP hydrolysis mechanism. Mol. Cell.

[40] Becke AD (1993). Density-functional thermochemistry. III. The role of exact exchange. J. Chem. Phys..

[41] Lee C, Yang W, Parr RG (1988). Development of the Colle-Salvetti correlation-energy formula into a functional of the electron density. Phys. Rev. B.

[42] Grimme S, Antony J, Ehrlich S, Krieg H (2010). A consistent and accurate ab initio parametrization of density functional dispersion correction (DFT-D) for the 94 elements H-Pu. J. Chem. Phys..

[43] Best RB (2012). Optimization of the additive CHARMM all-atom protein force field targeting improved sampling of the backbone *φ*, *ψ* and side-chain *χ*_1_ and *χ*_2_ dihedral angles. J. Chem. Theory Comput..

[44] Klamt A, Schüürmann G (1993). COSMO: a new approach to dielectric screening in solvents with explicit expressions for the screening energy and its gradient. J. Chem. Soc. Perkin Trans..

[45] Plessow P (2013). Reaction path optimization without NEB springs or interpolation algorithms. J. Chem. Theory Comput..

[46] Deglmann P, Furche F (2002). Efficient characterization of stationary points on potential energy surfaces. J. Chem. Phys..

[47] Kumar S, Bouzida D, Swendsen RH, Kollman PA, Rosenberg JM (1992). The weighted histogram analysis method for free-energy calculations on biomolecules. I. The method. J. Comput. Chem..

[48] Ahlrichs R, Bär M, Häser M, Horn H, Kölmel C (1989). Electronic structure calculations on workstation computers: the program system Turbomole. Chem. Phys. Lett..

[49] Brooks BR (2009). CHARMM: the biomolecular simulation program. J. Comput. Chem..

[50] Riahi S, Rowley CN (2014). The CHARMM-TURBOMOLE interface for efficient and accurate QM/MM molecular dynamics, free energies, and excited state properties. J. Comput. Chem..

[51] Prodromou C (1997). Identification and structural characterization of the ATP/ADP-binding site in the Hsp90 molecular chaperone. Cell.

[52] MacKerell AD (1998). All-atom empirical potential for molecular modeling and dynamics studies of proteins. J. Phys. Chem. B.

[53] Phillips JC (2005). Scalable molecular dynamics with NAMD. J. Comput. Chem..

[54] Humphrey W, Dalke A, Schulten K (1996). VMD: visual molecular dynamics. J. Mol. Graph..

[55] Baker NA, Sept D, Joseph S, Holst MJ, McCammon JA (2001). Electrostatics of nanosystems: application to microtubules and the ribosome. Proc. Natl. Acad. Sci. USA.

[56] Rabenstein B, Knapp E-W (2001). Calculated pH-dependent population and protonation of carbon-monoxy-myoglobin conformers. Biophys. J..

[57] Kieseritzky G, Knapp E-W (2008). Optimizing pK_A_ computation in proteins with pH adapted conformations. Proteins.

[58] Ramsey S, Orrell D, Bolouri H (2005). Dizzy: stochastic simulation of large-scale genetic regulatory networks. J. Bioinform. Comput. Biol..

[59] Tamura JK, Gellert M (1990). Characterization of the ATP binding site on *Escherichia coli* DNA gyrase. Affinity labeling of Lys-103 and Lys-110 of the B subunit by pyridoxal 5’-diphospho-5’-adenosine. J. Biol. Chem..

[60] Nathan DF, Lindquist S (1995). Mutational analysis of Hsp90 function: interactions with a steroid receptor and a protein kinase. Mol. Cell. Biol..

[61] Panjkovich A, Svergun DI (2018). CHROMIXS: automatic and interactive analysis of chromatography-coupled small-angle X-ray scattering data. Bioinformatics.

[62] Konarev PV, Volkov VV, Sokolova AV, Koch MHJ, Svergun DI (2003). *PRIMUS*: a Windows PC-based system for small-angle scattering data analysis. J. Appl. Cryst..

[63] Svergun DI (1992). Determination of the regularization parameter in indirect-transform methods using perceptual criteria. J. Appl. Cryst..

[64] Svergun D, Barberato C, Koch MHJ (1995). *CRYSOL* – a program to evaluate X-ray solution scattering of biological macromolecules from atomic coordinates. J. Appl. Cryst..

[65] Karagöz GE (2011). N-terminal domain of human Hsp90 triggers binding to the cochaperone p23. Proc. Natl. Acad. Sci. USA.

[66] Dehner A (2003). NMR chemical shift perturbation study of the N-terminal domain of Hsp90 upon binding of ADP, AMP-PNP, geldanamycin, and radicicol. ChemBioChem.

[67] Sattler M, Schleucher J, Griesinger C (1999). Heteronuclear multidimensional NMR experiments for the structure determination of proteins in solution employing pulsed field gradients. Prog. Nucl. Magn. Reson. Spectrosc..

[68] Delaglio F (1995). NMRPipe: a multidimensional spectral processing system based on UNIX pipes. J. Biomol. NMR.

[69] Vranken WF (2005). The CCPN data model for NMR spectroscopy: development of a software pipeline. Proteins Struct. Funct. Bioinf..

[70] Ikura M, Bax A, Clore GM, Gronenborn AM (1990). Detection of nuclear Overhauser effects between degenerate amide proton resonances by heteronuclear three-dimensional nuclear magnetic resonance spectroscopy. J. Am. Chem. Soc..

