# Supplementary material for: Conformational dynamics modulate the catalytic activity of the molecular chaperone Hsp90
Source: Nat Commun. 2020 Mar 16;11:1410. doi: 10.1038/s41467-020-15050-0 (PMC7075974; doi:10.1038/s41467-020-15050-0)
Supplement: Supplementary file 1 — Supplementary Information [file 41467_2020_15050_MOESM1_ESM.pdf]

Supplementary Material

Conformational dynamics modulate the catalytic activity of the molecular chaperone Hsp90

S. L. Mader et al.

Supplementary Content

Supplementary Figure 1. Overlap of the sampled reaction coordinates in the QM/MM umbrella sampling calculations.
Supplementary Figure 2. QM/MM reaction path profiles for ATP-hydrolysis in Hsp90 in different conformational states.
Supplementary Figure 3. Reaction path profiles and residue contributions with different residues excluded from the DFT models.
Supplementary Figure 4. DFT model of the wild type Hsp90 active site.
Supplementary Figure 5. Root-mean-square deviation (RMSD) of atomic positions in MD simulations.
Supplementary Figure 6. Root-mean-square fluctuation (RMSF) of atomic positions in MD simulations.
Supplementary Figure 7. Network of charged residues in the middle domain that stabilizes the open Arg-32/Glu33 ion-pair.
Supplementary Figure 8. Relative pK_a values of Glu-33 during 250 ns MD simulation of the full-length Hsp90 dimer.
Supplementary Figure 9. Duplicate MD simulations of the monomeric models.
Supplementary Figure 10. Reaction coordinates sampled during the ATP-hydrolysis reaction with an open Arg-32/Glu-33 ion-pair.
Supplementary Figure 11. Comparison of the structure and dynamics of the full-length wild type Hsp90 and the R32A variant from atomistic MD simulations.
Supplementary Figure 12. Interactions in the full-length wild type Hsp90 and Hsp90-R32A dimer in MD simulations.
Supplementary Figure 13. Chemical shift perturbations (CSP) of wild type NTD and the R32A variant.
Supplementary Figure 14. Fluorescence anisotropy measurements of unlabelled sba1-binding to wild type and R32A variants of yeast Hsp90.
Supplementary Figure 15. SAXS data of full-length Hsp90 wild type and the R32A variant.
Supplementary Figure 16. Plasmid shuffling experiments for yeast viability of the R32A variant as a sole source of Hsp90.
Supplementary Figure 17. K_M values for ATP binding in wild type Hsp90 and the R32A variant.
Supplementary Figure 18. ADP-release assay of the NM-fragment of wild type Hsp90 and the R32A variant.
Supplementary Figure 19. Formation of the closed FRET-dimer and ADP-release assay of Hsp90-E381Q.
Supplementary Figure 20. Kinetic model of the Hsp90 catalytic cycle.

Supplementary Table 1. Relative QM/MM reaction energies for ATP-hydrolysis in Hsp90.
Supplementary Table 2. Relative QM/MM reaction energies for ATP-hydrolysis in Hsp90 at different quantum chemical theory levels.
Supplementary Table 3. Vibrational zero-point energy and entropic contributions for phosphate dissociation estimated from DFT models.
Supplementary Table 4. Sidechain distances between Arg-32 and Glu-33 in different crystal structures.
Supplementary Table 5. Ratio of apparent closing and re-opening rates of wild type Hsp90 and the R32A variant obtained from FRET measurements.
Supplementary Table 6. Summary of SAXS data.
Supplementary Table 7. Data-collection and scattering-derived parameters for SAXS data.
Supplementary Table 8. Rate coefficients employed for kinetic models.
Supplementary Table 9. Residue numbering in different Hsp90 species.
Supplementary Table 10. Summary of all molecular simulations.
Supplementary Table 11. Primer sequences of the different constructs.

Supplementary References

Supplementary figures

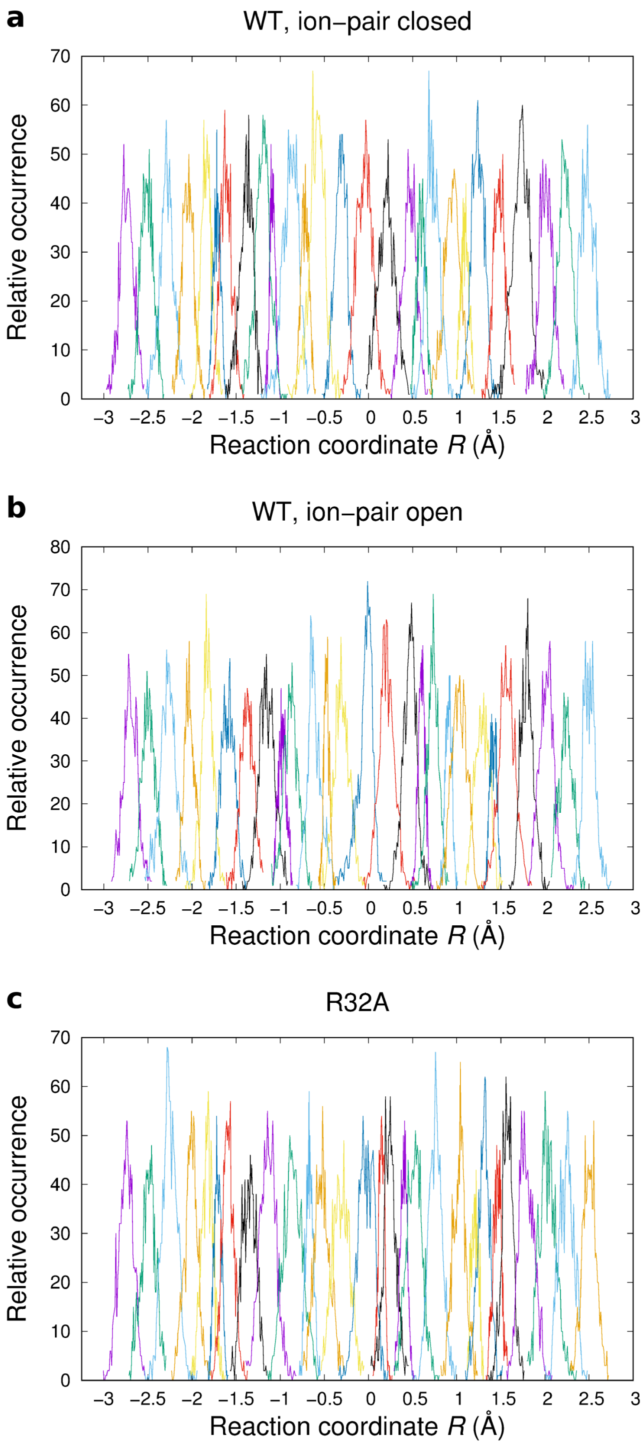

Supplementary Figure 1. Overlap of the reaction coordinate in the QM/MM umbrella sampling calculations. The different colours are used to highlight the individual umbrella windows.

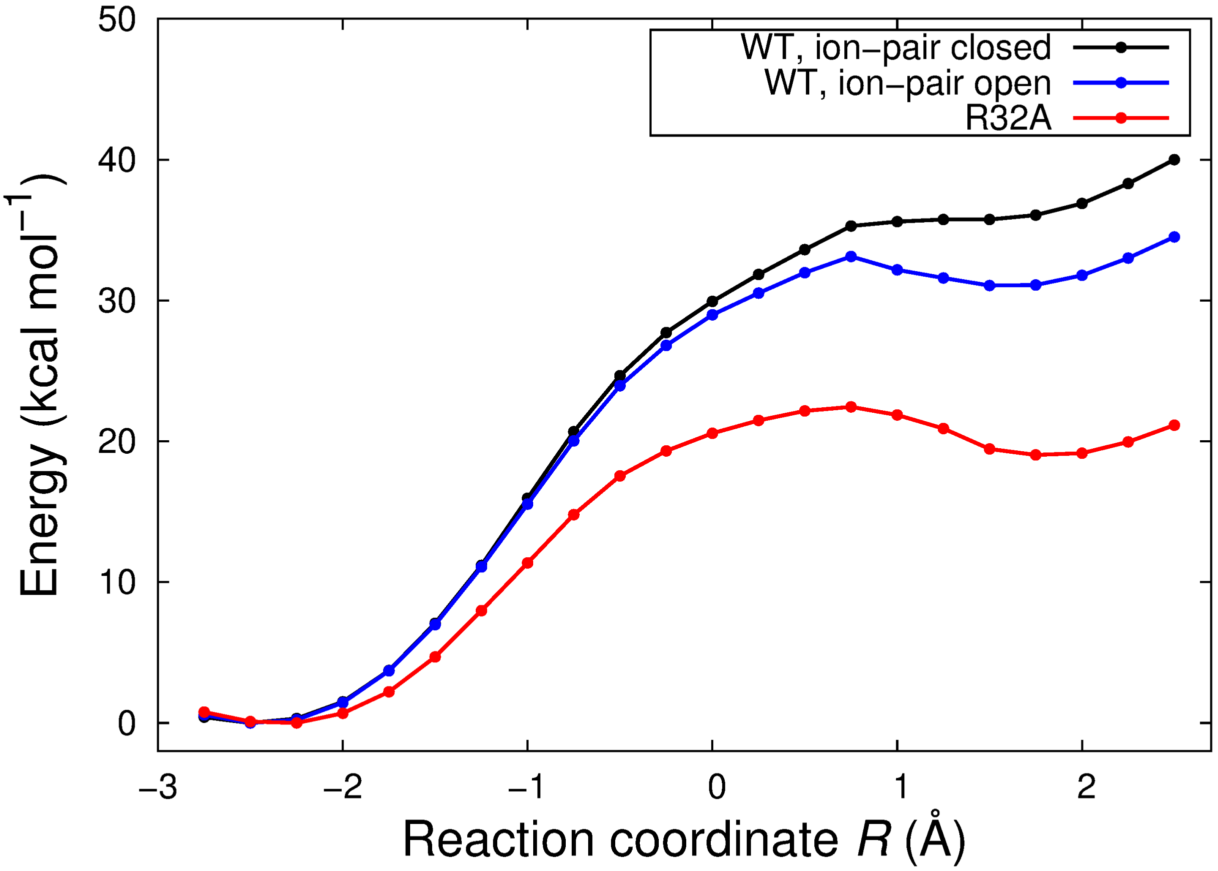

Supplementary Figure 2. QM/MM reaction path optimizations for ATP hydrolysis in Hsp90 with the R32/E33 ion pair closed (in black) and open (in blue), as well as for the R32A variant (in red).

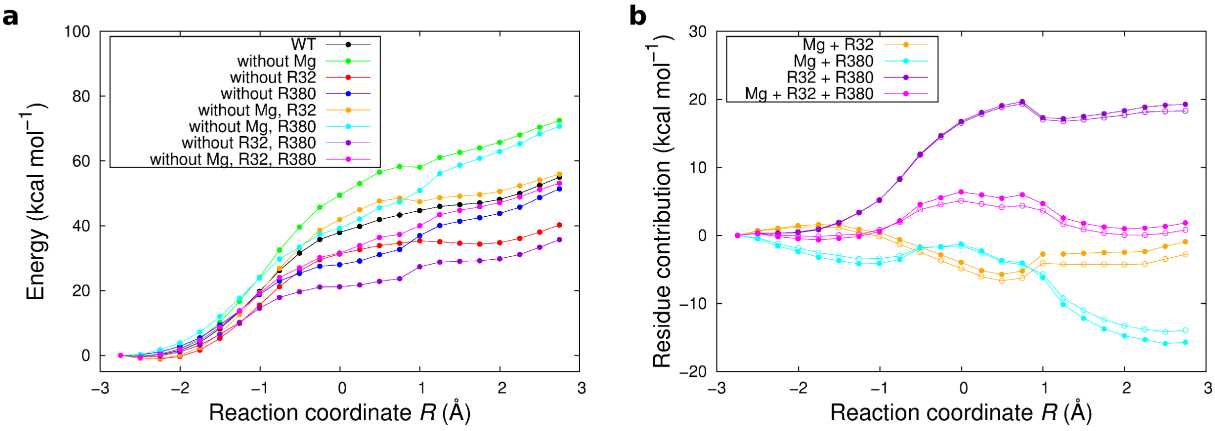

Supplementary Figure 3. a) Single point calculations of the reaction path structures of wild type Hsp90 with the R32/E33 ion-pair closed (WT) and with different residues excluded from the DFT models. **b)** Energy difference relative to the wild type calculation (panel **a**, black trace), obtained by switching off multiple residue contributions. Values plotted with filled circles are obtained from calculations with multiple residues removed from the models. Values plotted with empty circles are calculated from adding up the contributions of the individual residues (see main Fig. 3a).

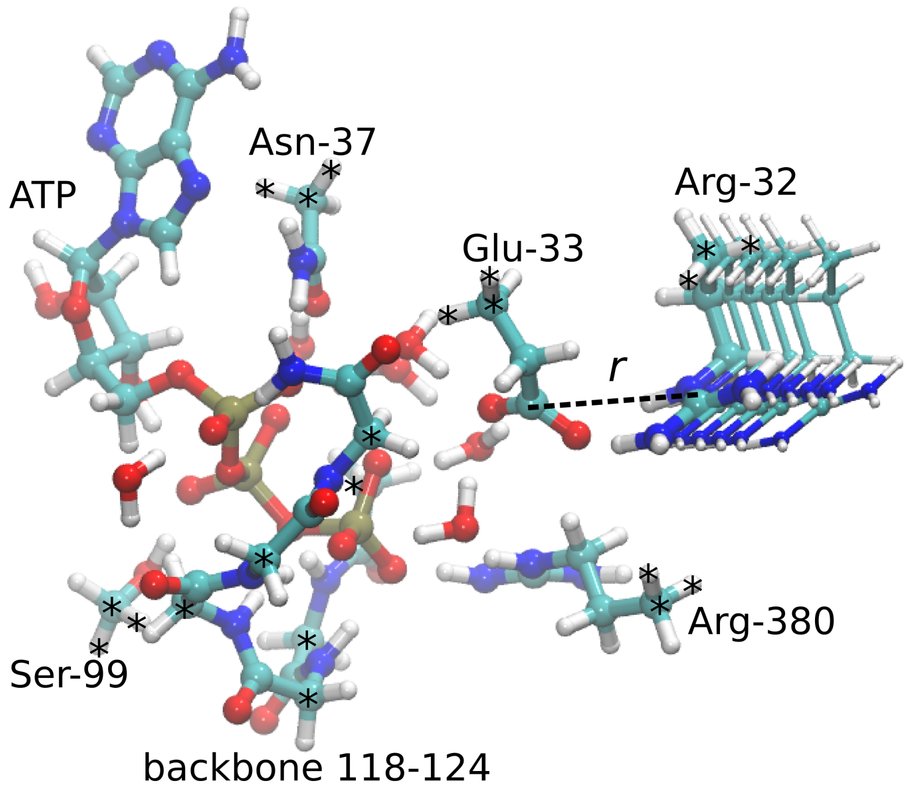

Supplementary Figure 4. DFT model of the wild type Hsp90 active site. Terminal carbon and hydrogen atoms that were kept fixed during structure optimization are marked with an asterisk. The Arg-32 residue was gradually displaced from Glu-33 to estimate the electrostatic tuning effect.

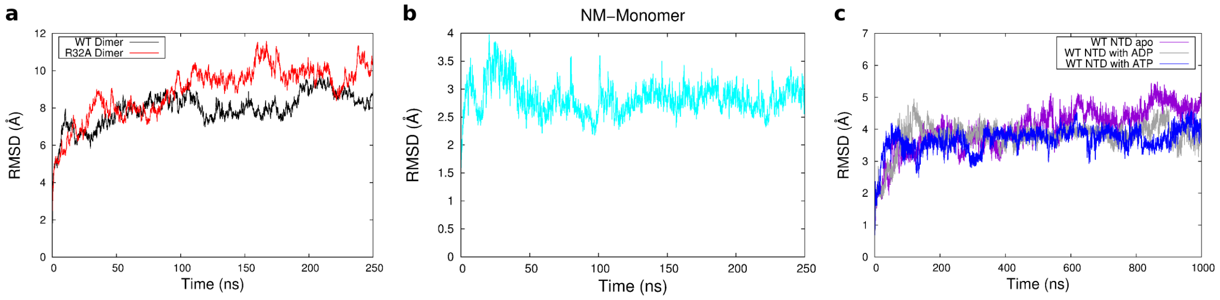

Supplementary Figure 5. Root-mean-square deviation (RMSD) of atomic positions in MD simulations of **a)** full-length Hsp90 dimers, starting from the crystal structure PDB ID: 2CG9,[1] **b)** monomeric wild type NM-domain model, built from the crystal structure PDB ID: 4IVG,[2] and **c)** NTD models, starting from the crystal structure PDB ID: 1AMW.[3]

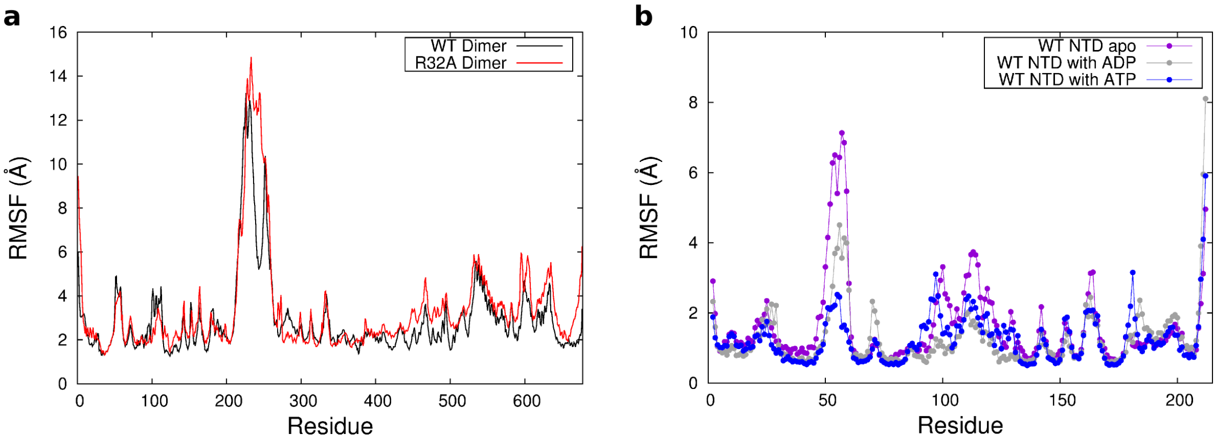

Supplementary Figure 6. Root-mean-square fluctuations (RMSF) of atomic positions in MD simulations of **a)** full-length Hsp90 dimers, starting from the crystal structure PDB ID: 2CG9,[1] and **b)** NTD models, starting from the crystal structure PDB ID: 1AMW.[3]

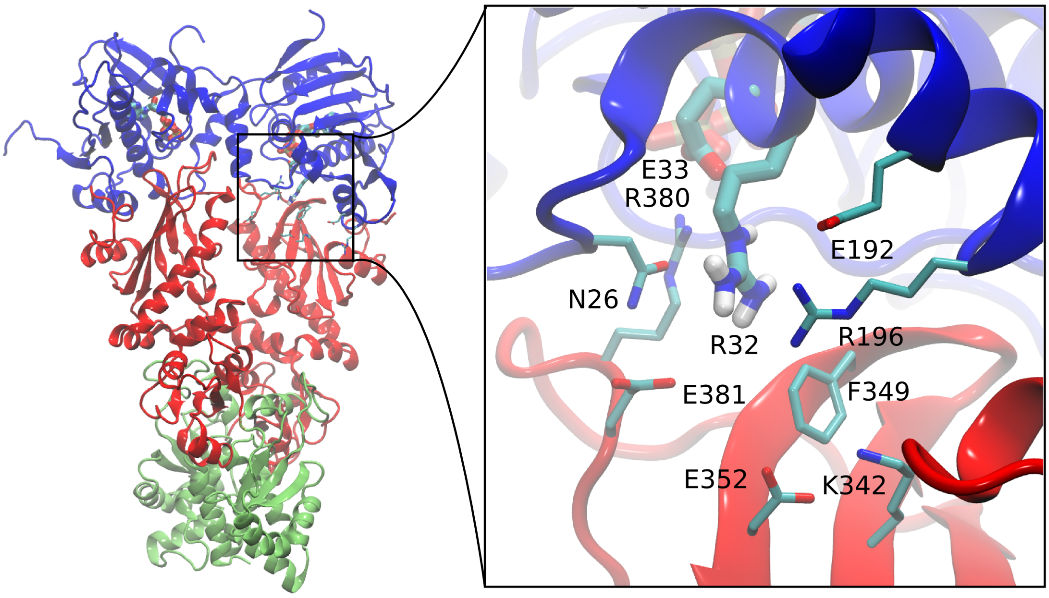

Supplementary Figure 7. The open Arg-32/Glu33 ion pair is stabilized by a network of charged residues of the middle domain. The structure shown is a snapshot from a 250 ns MD simulation of the full length Hsp90.

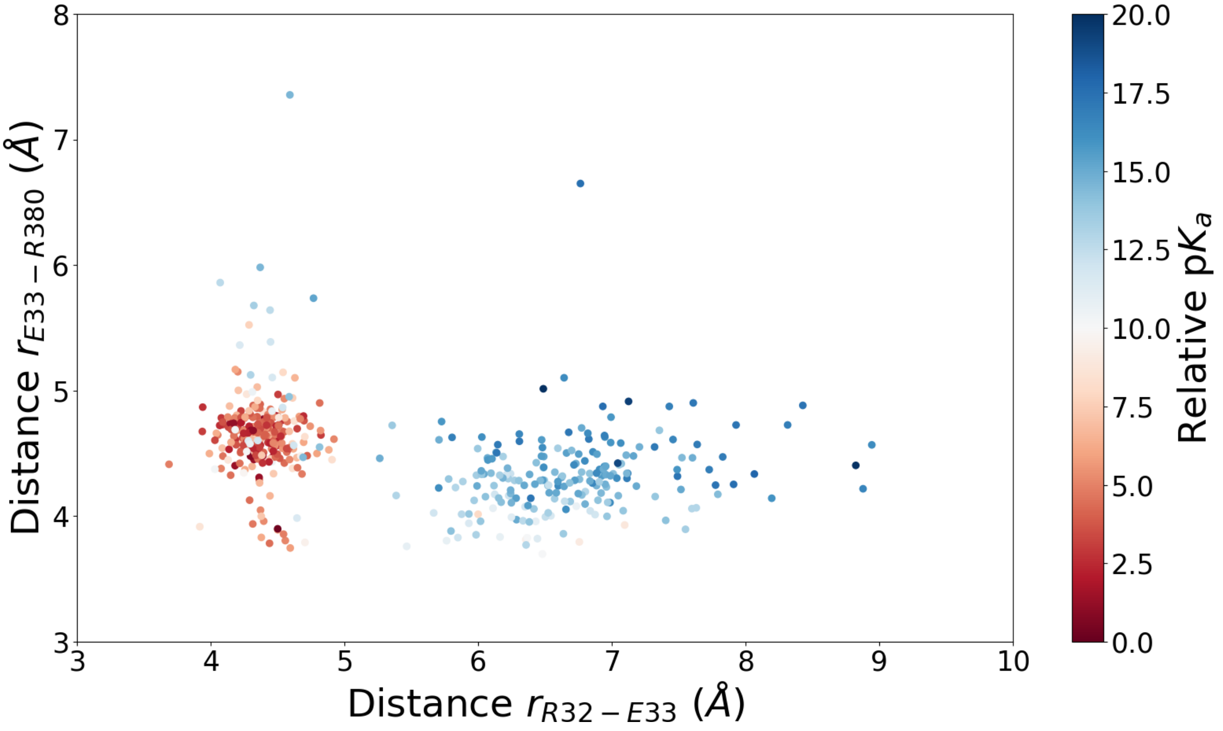

Supplementary Figure 8. Relative pK_a values of Glu-33 during 250 ns MD simulation of the full-length Hsp90 dimer.

.

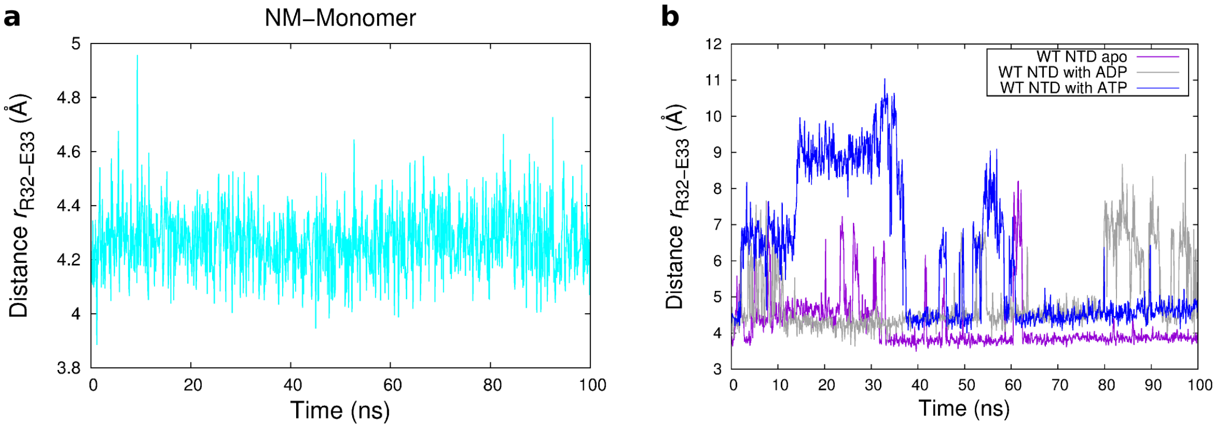

Supplementary Figure 9. Side chain distance between Arg-32 and Glu-33 in duplicate MD simulations of **a)** monomeric wild type NM-domain model and **b)** wild type NTD models.

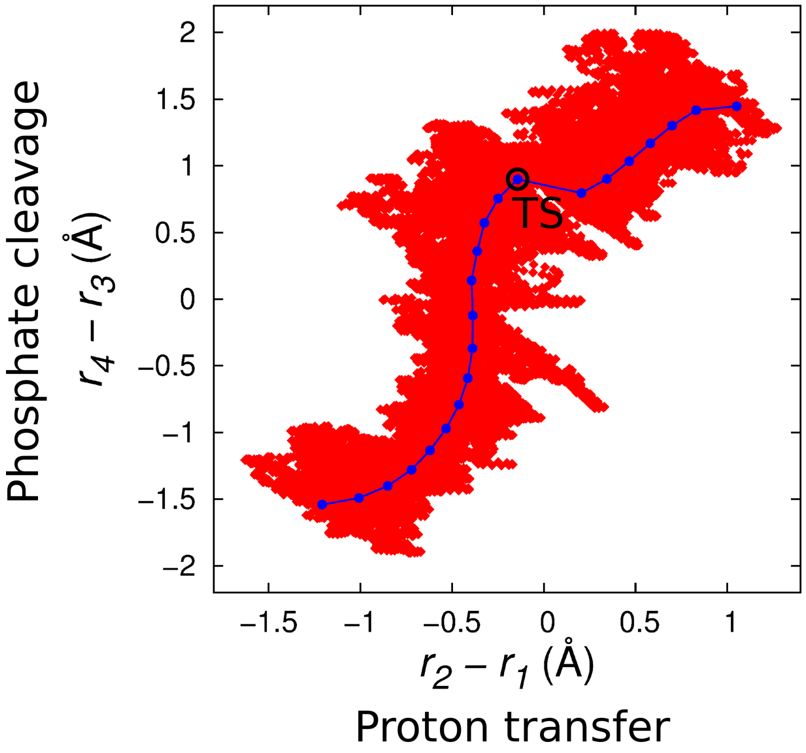

Supplementary Figure 10. Semi-concerted ATP-hydrolysis mechanism from QM/MM free energy calculations (red dots) and reaction path optimization (blue dots), showing the sampled reaction coordinate with the Arg-32/Glu-33 ion-pair open. The reaction coordinate used for the QM/MM calculations is $R = r_4 - r_3 + r_2 - r_1$, a linear combination of distances between Glu-33, the attacking water molecule, and the γ-phosphate group of ATP (see main Fig. 2b). The transition state (TS) of the reaction path optimization is marked with a black circle.

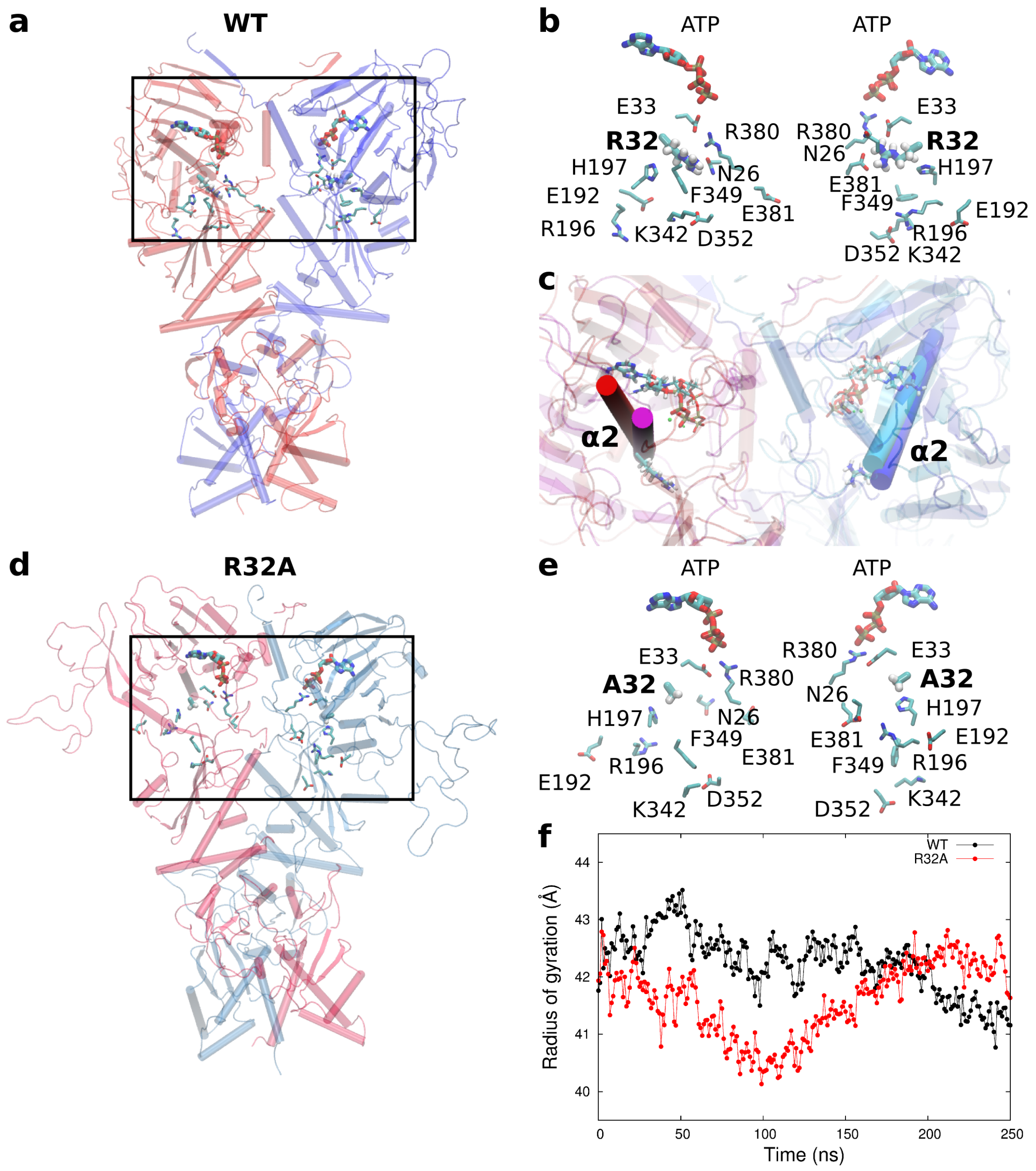

Supplementary Figure 11. Comparison of the structure and dynamics of the full-length wild type (WT) Hsp90 (panels **a** and **b**) and the R32A variant (**d** and **e**) from atomistic MD simulations. Close-up of the active sites are shown in panels **b** and **e** after 250 ns MD simulations. In panel **c** is overlaid the wild type (red, blue) and the R32A variant (pink, cyan) after 250 ns MD simulations, showing conformational changes in helix α2. **f)** Radius of gyration calculated from 250 ns MD simulation of wild type (WT) Hsp90 (in black) and the R32A variant (in red) starting from the crystal structure of the closed Hsp90 dimer (PDB ID: 2CG9).[1]

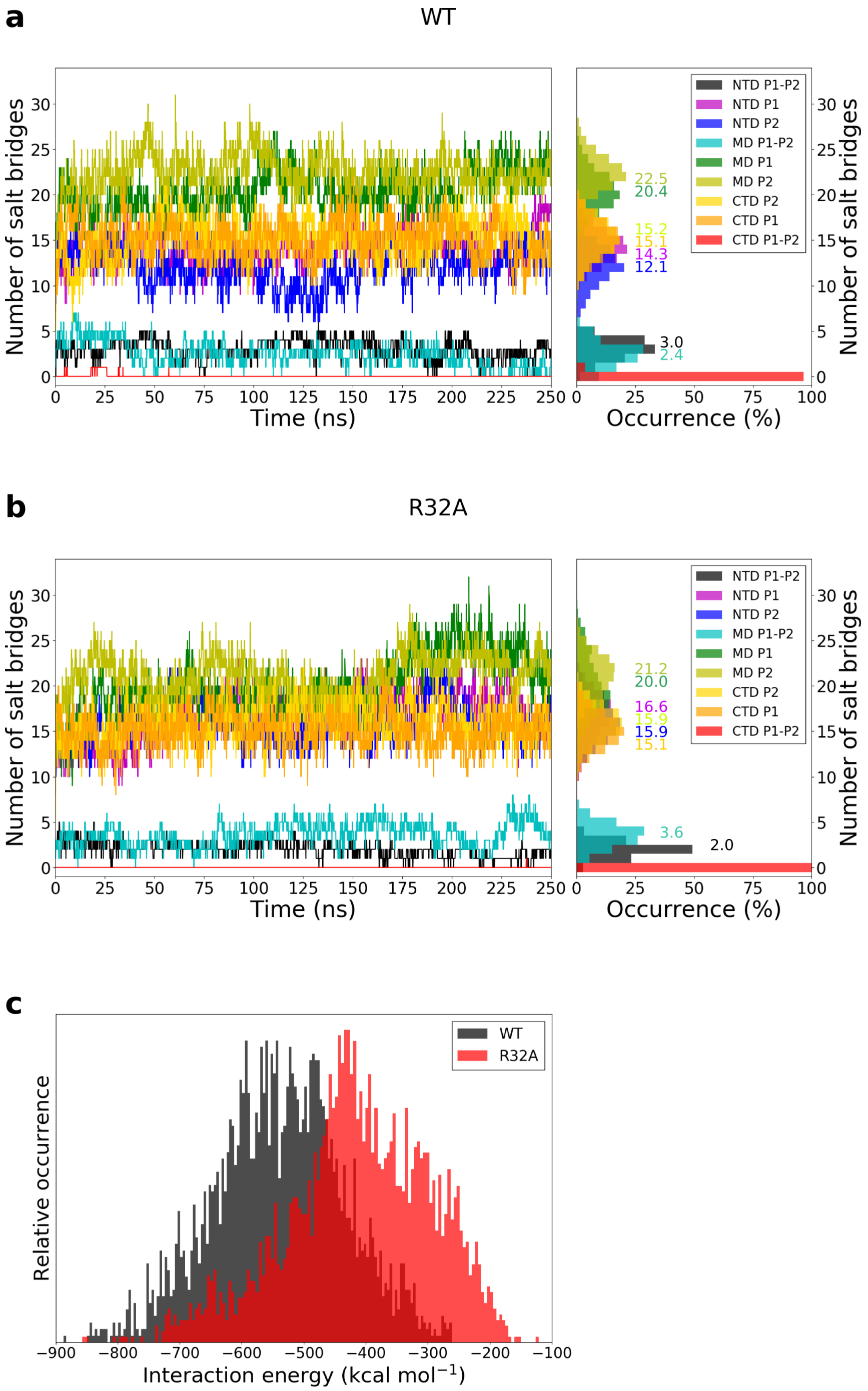

Supplementary Figure 12. Interactions in the full-length wild type (WT) Hsp90 and Hsp90-R32A dimers in MD simulations. Intra- and inter-domain ion-pairs in the two protomers (P1, P2) of **a)** the full-length wild type (WT) Hsp90 dimer and **b)** the Hsp90-R32A variant during 250 ns MD simulation. **c)** Interaction energy between the two N-terminal domains of the full-length dimer of wild type (WT) Hsp90 (in black) and the R32A variant (in red) during the MD simulation shows a weaker interaction energy for the R32A variant.

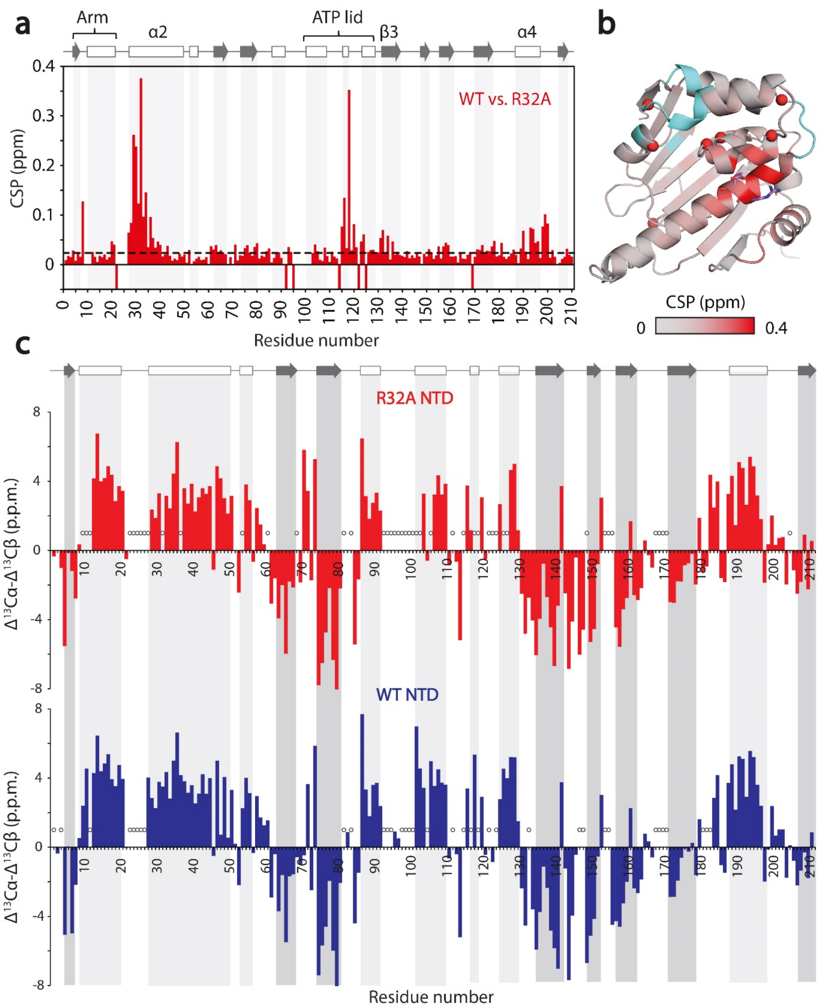

Supplementary Figure 13. a) Chemical shift perturbations (CSP) of wild type NTD and the R32A variant. Negative bars represent residues that could not be assigned due to unfavourable conformational exchange. Secondary structure elements are shown on the top. **b)** Distribution of CSP in the crystal structure of the N-terminal domain. Amide groups of residues that are not visible due to increased conformational dynamics in the R32A mutant are represented by red spheres, and unassigned residues are coloured in cyan. **c)** Secondary ^{13}C chemical shifts of the R32A NTD (top, red) as a function of the residue number, as compared to the wild type NTD (bottom, blue). Secondary chemical shifts were calculated as the difference between the experimental ^{13}C shift and its random coil shift.[4] Positive values indicate an α-helical conformation, negative values indicate β-strands, and zero values indicate unstructured regions. Structural elements extracted from the TALOS+ software[5] are shown on the top: Arrows represent β-strands, rectangles represent α-helices, and empty dots indicate residues without data. The high similarity between the secondary ^{13}C shifts of the wild type and the R32A variant confirm the integrity of the secondary structure.

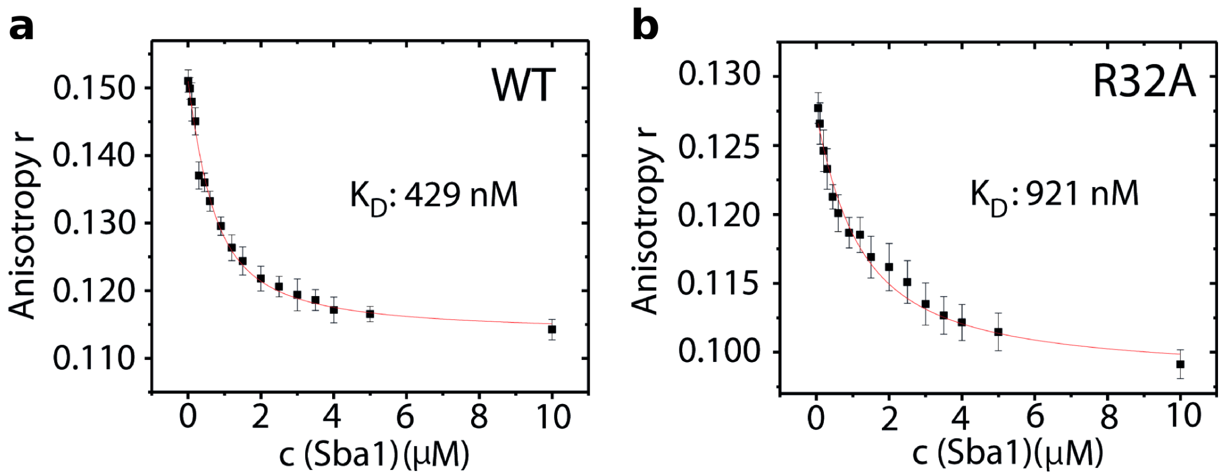

Supplementary Figure 14. Fluorescence anisotropy measurements and K_D-values of unlabelled sba1 binding to **a)** wild type yeast Hsp90 and **b)** the R32A variant. Sba1 is a co-chaperone stabilising the closed conformation of Hsp90.[6] After pre-incubation of Hsp90 with labelled sba1, unlabelled sba1 was added in increasing concentrations to compete out the preformed labelled sba1-Hsp90 complex. A mono-exponential decay equation was used for fitting. Data-points represent a mean of 15 measurements from a single sample. Error bars represent SD of 15 measurements from a single sample (n = 15). Source data are provided as a Source Data file.

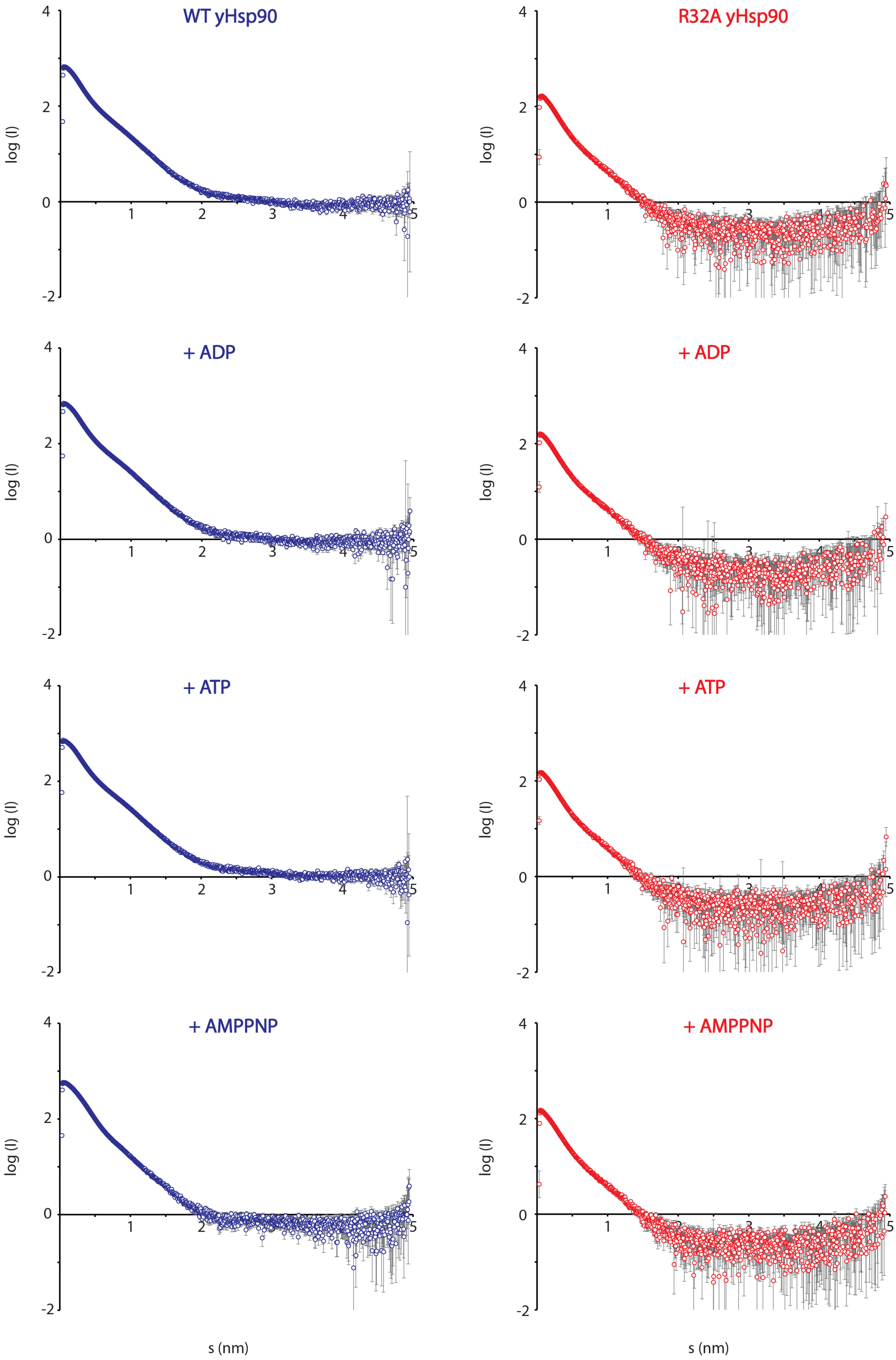

Supplementary Figure 15. Scattering profiles (log *I vs. s*, where *I* is the scattering intensity, and *s* is the modulus of the momentum transfer) of the full-length wild type (WT, in blue) and R32A variant (in red) of Hsp90 in different nucleotide states. Data shown correspond to the averaged profiles for the selected frames of the chromatographic peak.

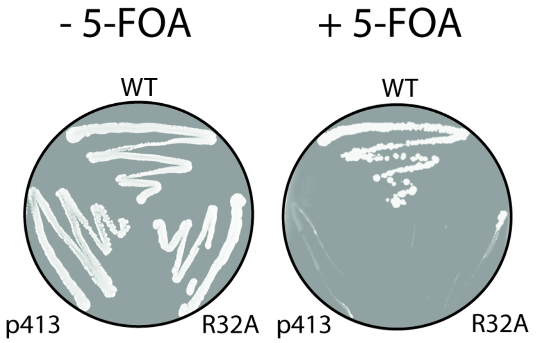

Supplementary Figure 16. Plasmid shuffling experiments for yeast viability of the R32A variant as a sole source of Hsp90. The shuffling strain ΔPCLDα was transformed with the p413-GPD containing the R32A variant, wild type Hsp82 (positive control), or the empty p413-GPD (negative control), on media without (left) and with (right) 5-fluoroorotic acid (5-FOA).

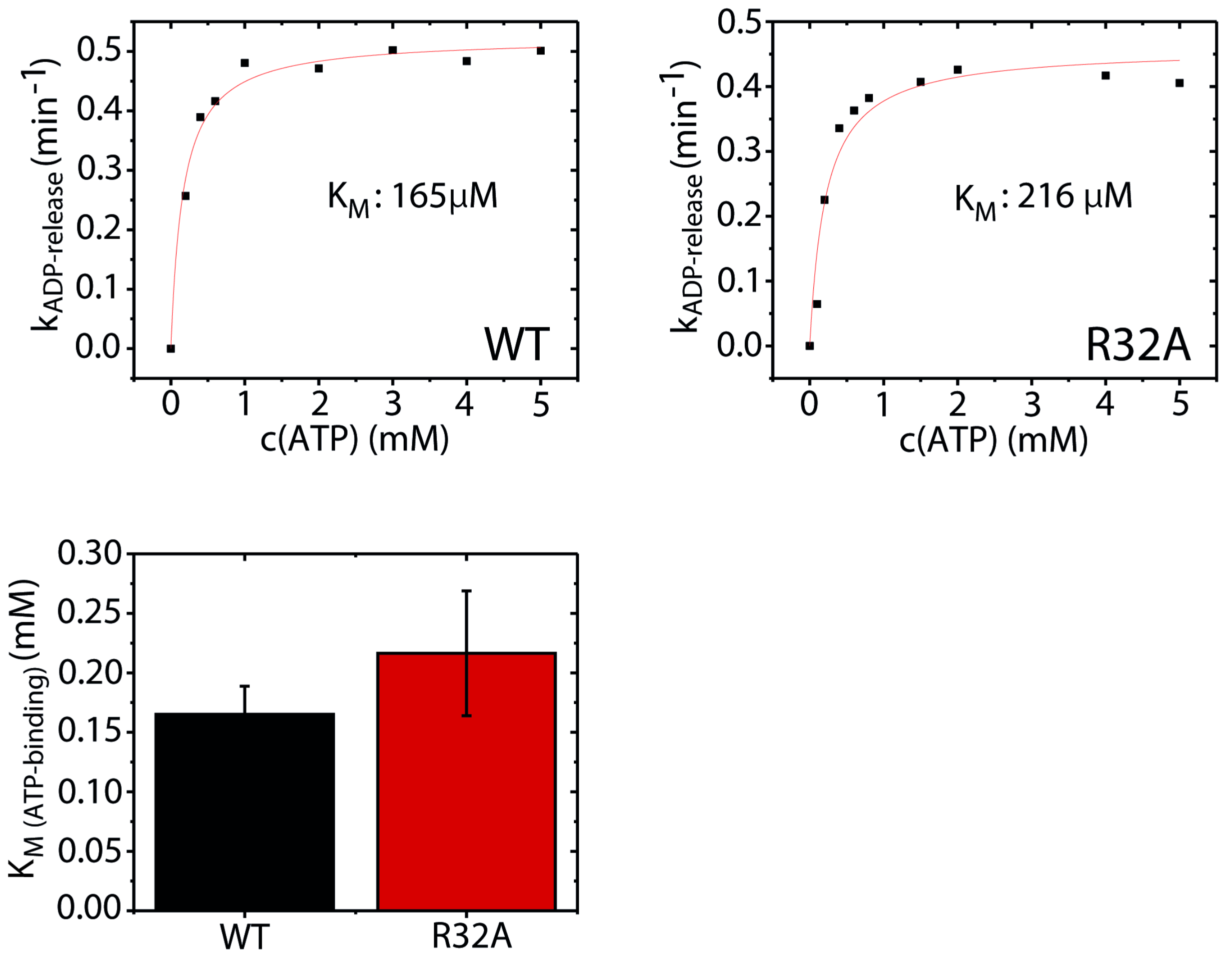

Supplementary Figure 17. K_M values for ATP binding in wild type (WT) Hsp90 and the R32A variant. ADP-release assays were performed with increasing concentrations of ATP. Data were fitted using the Michaelis-Menten equation. Error bars represent SD of the individual data points from the calculated fit for a single measurement ($n = 1$). Source data are provided as a Source Data file.

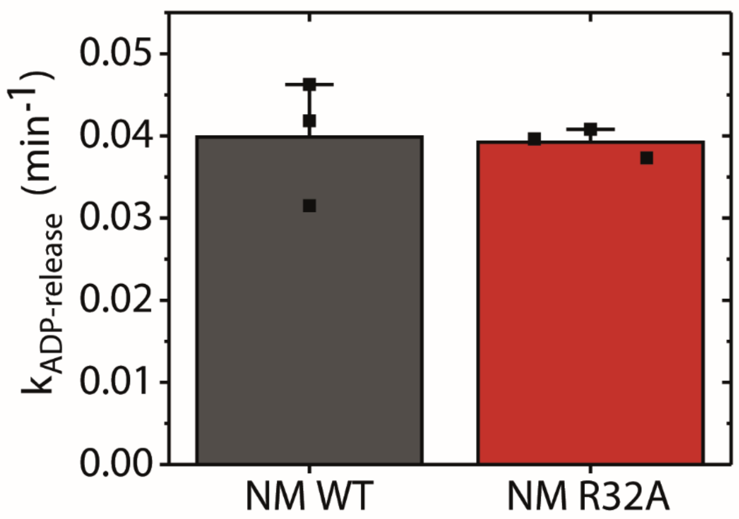

Supplementary Figure 18. ADP-release assay of the NM-fragment of wild type Hsp90 and the R32A variant. Error bars represent SD from three independent measurements ($n = 3$), shown as black dots. Source data are provided as a Source Data file.

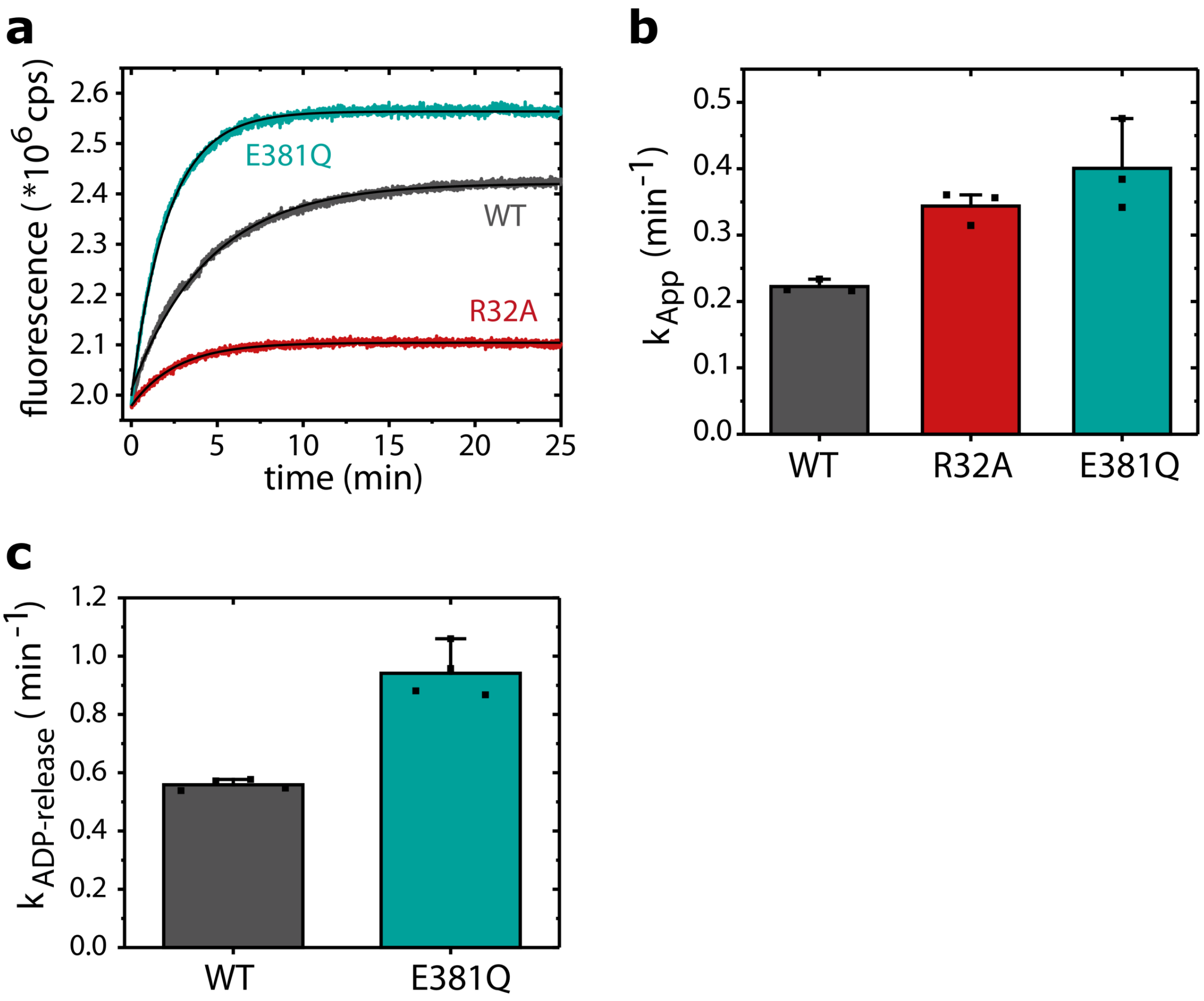

Supplementary Figure 19. a) FRET experiments of WT Hsp90, and the R32A and E381Q variants, where formation of the N-terminally closed Hsp90 dimers is induced by addition of ATPγS. **b**) Closing rates for WT, R32A, and E381Q obtained from mono-exponential fitting of the FRET curves shown in **a**). Error bars represent SD from three independent measurements ($n = 3$), shown as black dots. **c**) ADP-release rates of WT Hsp90 and the E381Q variant. Error bars represent SD from four independent measurements ($n = 4$), shown as black dots. Source data are provided as a Source Data file.

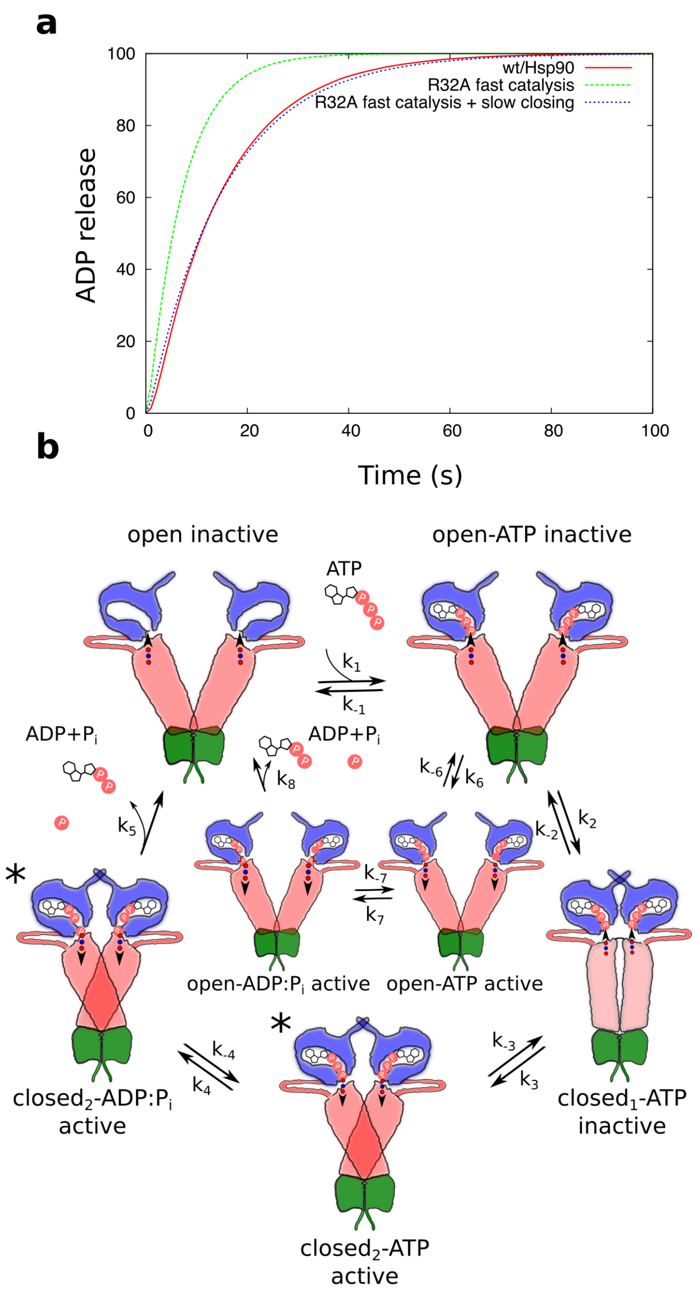

Supplementary Figure 20. Kinetic model of the Hsp90 catalytic cycle. **a)** Kinetic simulations of the wild type Hsp90 (in red), the R32A model with an increased catalytic rate (in green), and the R32A model with an increased catalytic rate and decreased closing population (in blue). The fully decoupled cycle (k_6/k_{-6}-k_8) was not modelled in the shown simulation, as the elementary rates are unknown. **b)** Kinetic model of the Hsp90 catalytic cycle. Kinetic parameters are given in Supplementary Table 8.

Supplementary Tables

Supplementary Table 1. Relative QM/MM reaction energies (in kcal mol^{-1}) for ATP-hydrolysis in Hsp90 with def2-SVP basis sets (def2-TZVP for Mg) and def2-TZVP basis sets.

	WT, R32/E33 closed B3LYP/SVP (kcal mol^{-1})	WT, R32/E33 closed B3LYP/TZVP (kcal mol^{-1})	WT, R32/E33 open B3LYP/SVP (kcal mol^{-1})	WT, R32/E33 open B3LYP/TZVP (kcal mol^{-1})	R32A variant B3LYP/SVP (kcal mol^{-1})	R32A variant B3LYP/TZVP (kcal mol^{-1})
Reactant state	0	0	0	0	0	0
Transition state	35.8	36.3	33.1	35.2	22.5	24.5
Product state	35.8	35.8	31.1	31.8	19.0	19.1

Supplementary Table 2. Relative QM/MM reaction energies (in kcal mol^{-1}) for the ATP-hydrolysis reaction in the R32A variant of Hsp90 calculated with different density functionals. All calculations were performed with def2-SVP on all atoms except for Mg (def2-TZVP).

**Method**	**ATP state** (kcal mol^{-1})	**Transition state** (kcal mol^{-1})	**ADP+P_i state** (kcal mol^{-1})
B3LYP-D3	0	22.5	19.0
B3LYP*-D3 (15% exchange)	0	21.7	18.8
BHLYP-D3	0	25.8	20.2
TPSSh-D3	0	21.8	18.5
BP86-D3	0	19.1	17.7

Supplementary Table 3. Vibrational zero-point energy (ΔZPE) and entropic ($T\Delta S$) contributions for phosphate dissociation estimated from DFT models.

Reaction	ΔZPE (kcal mol^{-1})	$T\Delta S$ (kcal mol^{-1})	ΔZPE – $T\Delta S$ (kcal mol^{-1})
ADP…P_i + GluH → ADP + GluH + P_i (bulk)	-0.3	16.4	-16.7

Supplementary Table 4. Sidechain distances between Arg-32 and Glu-33 in different crystal structures. Open ion-pairs are highlighted in bold font.

PDB ID	Description	Sidechain distance R32-E33
1A4H	Yeast, NTD, with Geldanamycin	4.43 Å
1AH6	Yeast, NTD, *apo*	4.35 Å
1AH8, chain A	Yeast, NTD, *apo*	**7.00 Å**
1AH8, chain B		4.96 Å
1AM1	Yeast, NTD, with ATP	**6.09 Å**
1AMW	Yeast, NTD, with ADP	4.27 Å
2WEP	Yeast, NTD:L34I:I35V, with ADP	4.08 Å
2CG9, chain A	Yeast, full-length dimer, with AMP-PNP	4.35 Å
2CG9, chain B		4.38 Å
3T0Z (residues 46/47)	Human, NTD, with ATP	4.59 Å
4IVG (residues 129/130)	*D. rerio*, NTD:MD, with AMP-PNP	4.36 Å
4IPE, chain A	*D. rerio,* full-length dimer, with AMP-PNP	4.28 Å
4IPE, chain B		4.37 Å
5ULS, chain A (residues 102/103)	GRP94, full-length dimer, with AMP-PNP	4.07 Å
5ULS, chain B		4.48 Å

Supplementary Table 5. Ratio of apparent closing and re-opening rates of wild type Hsp90 and the R32A variant obtained from FRET measurements.

	**WT** (min^{-1})	**R32A** (min^{-1})
Closing	0.223 ± 0.01	0.348 ± 0.025
Re-opening	0.062 ± 0.005	0.873 ± 0.083
Ratio	3.584	0.394

Supplementary Table 6. Summary of SAXS data. R_g values are obtained from the Guinier approximation, and $P(r)$ distribution functions from the Gnom software.[7]

**State**	**WT** (in nm)	**R32A** (in nm)
Apo	R_g 6.23 ± 0.01<br>D_{max} 24.6	R_g 6.55 ± 0.02<br>D_{max} 29.2
ADP	R_g 5.94 ± 0.01<br>D_{max} 22.4	R_g 6.88 ± 0.05<br>D_{max} 29.9
ATP	R_g 5.76 ± 0.08<br>D_{max} 24.5	R_g 6.55 ± 0.03<br>D_{max} 27.7
AMPPNP	R_g 5.31 ± 0.01<br>D_{max} 26.2	R_g 6.23 ± 0.03<br>D_{max} 29.2

Supplementary Table 7. Data-collection and scattering-derived parameters for SAXS data.

**Data collection parameters**	Apo-WT	ADP-WT	ATP-WT	AMPPNP-WT	Apo-R32A	ADP-R32A	ATP-R32A	AMPPNP-R32A
Instrument	ESRF BM29	ESRF BM29	ESRF BM29	ESRF BM29	ESRF BM29	ESRF BM29	ESRF BM29	ESRF BM29
Beam geometry (mm^2)	0.7 × 0.7	0.7 × 0.7	0.7 × 0.7	0.7 × 0.7	0.7 × 0.7	0.7 × 0.7	0.7 × 0.7	0.7 × 0.7
Wavelength (Å)	0.992	0.992	0.992	0.992	0.992	0.992	0.992	0.992
q range ($Å^{-1}$)	0.003-0.495	0.003-0.495	0.003-0.495	0.003-0.495	0.003-0.495	0.003-0.495	0.003-0.495	0.003-0.495
Exposure time (s, per frame)	1	1	1	1	1	1	1	1
Concentration range (mg ml^{-1}, injected sample)	46	46	46	46	5.2	5.2	5.2	5.2
Temperature (K)	293	293	293	293	293	293	293	293
**Structural parameters**								
$I(0)$ (cm^{-1}) [from $P(r)$]	707 ± 0.4	731 ± 0.6	759 ± 0.7	610 ± 1	180 ± 1	166 ± 1	158 ± 1	151 ± 1
R_g (Å) [from $P(r)$]	64.8 ± 0.07	62.1 ± 0.06	62.7 ± 0.1	57.6 ± 0.03	69.7 ± 0.7	73.3 ± 0.7	71.6 ± 0.6	69.1 ± 1
$I(0)$ (cm^{-1}) [from Guinier]	700 ± 0.9	721 ± 0.7	733 ± 0.8	595 ± 0.8	177 ± 0.4	164 ± 0.5	153 ± 0.5	145 ± 0.4
R_g (Å) [from Guinier]	62.3 ± 0.1	59.4 ± 0.1	57.6 ± 0.8	53.1 ± 0.1	65.5 ± 0.2	68.8 ± 0.5	65.5 ± 0.3	62.3 ± 0.3
D_{max} (Å)	246	224	245	262	292	299	277	292
Porod volume estimate ($Å^3$)	366680	339334	330672	311435	457074	523573	443394	419298
Dry volume calculated from sequence ($Å^3$)	198782	198782	198782	198782	198575	198575	198575	198575
**Molecular mass determination**								
Molecular mass M_r [Rambo and Tainer, Nature, 496, 477–481, 2013]	169727	158358	152221	181892	204065	204606	198653	192696
Calculated dimeric M_r from sequence	162812	163239	163319	16318	162642	163069	163149	163148
**Software employed**								
Primary data reduction	BsxCuBE EDNA	BsxCuBE EDNA	BsxCuBE EDNA	BsxCuBE EDNA	BsxCuBE EDNA	BsxCuBE EDNA	BsxCuBE EDNA	BsxCuBE EDNA
Data processing	CHROMIXS, PRIMUS	CHROMIXS, PRIMUS	CHROMIXS, PRIMUS	CHROMIXS, PRIMUS	CHROMIXS, PRIMUS	CHROMIXS, PRIMUS	CHROMIXS, PRIMUS	CHROMIXS, PRIMUS
Distance distribution	GNOM	GNOM	GNOM	GNOM	GNOM	GNOM	GNOM	GNOM

Supplementary Table 8. Rate coefficients (s^{-1}) employed for kinetic simulations of the Hsp90 cycle. The kinetic simulations were performed in Dizzy,[8] using the Gillespie algorithm with an ensemble size of 1000. Kinetic parameters for Hsp90 were estimated from ref. 9. The approximate ATPase reaction rate (k_4) for R32A was estimated based on quantum chemical calculations. A perturbed closing rate, k_3=0.3 s^{-1}, gives an unperturbed rate for the ADP release reaction. The fully decoupled cycle (k_6/k_{-6}-k_8) was not kinetically modelled, as the elementary rates are unknown.

	k_1	k_{-1}	k_2	k_{-2}	k_3	k_{-3}	k_4	k_{-4}	k_5
Wild type	40	8.7	0.4	1.5	**1.0**	0.8	**0.6**	**0.003**	20
R32A mutant	40	8.7	0.4	1.5	**0.3**	0.8	**90**	**0.03**	20

Supplementary Table 9. Residue numbering of Hsp90 in yeast and *Danio rerio*.

Yeast	Arg-32	Glu-33	Asn-37	Ser-99	GQFGVGF 118-124	Phe-349	Arg-380	Glu-381
Danio rerio	Arg-129	Glu-130	Asn-134	Ser-193	GQFGVGF 214-220	Phe-401	Arg-417	Glu-418

Supplementary Table 10. Summary of molecular simulations.

Simulation number	Model	Method	System size	Simulation length
1	Full length WT, *w.* ATP	MD	303,000	250 ns
2	Full length, R32A, *w.* ATP	MD	303,000	250 ns
3	NM-model, WT *w.* ATP	MD	72,300	250 ns, 100 ns (duplicate)
4	NTD-model, WT *w.* ATP	MD	77,300	1000 ns, 100 ns (duplicate)
5	NTD-model, WT *w.* ADP	MD	77,300	1000 ns, 100 ns (duplicate)
6	NTD-model, WT *apo*	MD	77,300	1000 ns, 100 ns (duplicate)
7	NTD-model, R32A *w.* ATP	MD	77,300	1000 ns
8	NTD-model, R32A *w.* ADP	MD	77,300	1000 ns
9	NTD-model, R32A *apo*	MD	77,300	1000 ns
10	Trimmed N/M/ATP, WT, R32/E33 ion-pair closed	QM/MM free energy	17,000	27 ps
11	Trimmed N/M/ATP, WT, R32/E33 ion-pair open	QM/MM free energy	17,000	27 ps
12	Trimmed N/M/ATP, R32A	QM/MM free energy	17,000	27 ps
13	Trimmed N/M/ATP, WT, R32/E33 ion-pair closed	QM/MM reaction pathway	17,000	*N/A*
14	Trimmed N/M/ATP, WT, R32/E33 ion-pair open	QM/MM reaction pathway	17,000	*N/A*
15	Trimmed N/M/ATP, R32A	QM/MM reaction pathway	17,000	*N/A*
16	Active site region	DFT cluster	169	*N/A*

Supplementary Table 11. Primer sequences for the different constructs of yeast Hsp90 (Hsp82).

Construct	Primer sequences
R32A single point mutation	5'-AATTTTCTTGGCGGAACTGATATCTAATGCC-3'<br>5'-TCCTTGTTAGAATAGACG-3'
E381Q single point mutation	5'-TTTGTCCAGACAGATGTTACAACAAAATAAG-3'<br>5'-TTCAATGGTAAATCCTCAG-3'
D61C single point mutation (for FRET)	5'-AACAGAACCATGCCTCTTTATTAGAATCACTC-3'<br>5'-TCCAATTGCTTTGGATCAG-3'
NM-domains	5'-GATCTGCTCGAGTTATTCGAAATCTTTAGTAA TGTCAACCAAAG-3'<br>5'-GATCTCGGATCCCGCGGCATGGCCAGTGAAA CTTTTGAATTTC-3'
N-terminal domain	5'-GATCTCCCATGGCGCGGCATGGCTAGTGAAA CTTTTGAATTTC-3'<br>5'-GATCTGCTCGAGTTAGGTGACGACTAATTGG ATTGGGTAGGCC-3'

Supplementary references

1. Ali, M. M. U. et al. Crystal structure of an Hsp90-nucleotide-p23/Sba1 closed chaperone complex. *Nature* **440**, 1013–1017 (2006).
2. Lavery, L. A. et al. Structural Asymmetry in the Closed State of Mitochondrial Hsp90 (TRAP1) Supports a Two-Step ATP Hydrolysis Mechanism. *Mol. Cell* **53**, 330–343 (2014).
3. Prodromou, C. et al. Identification and Structural Characterization of the ATP/ADP-Binding Site in the Hsp90 Molecular Chaperone. *Cell* **90**, 65–75 (1997).
4. Wishart, D. S. et al. ^{1}H, ^{13}C and ^{15}N chemical shift referencing in biomolecular NMR. *J. Biomol. NMR* **6**, 135-140 (1995).
5. Shen, Y., Delaglio, F., Cornilescu, G. & Bax, A. TALOS+: a hybrid method for predicting protein backbone torsion angles from NMR chemical shifts. *J. Biomol. NMR* **44**, 213-223 (2009).
6. Schopf, F. H., Biebl, M. M. & Buchner, J. The HSP90 chaperone machinery. *Nat. Rev. Mol. Cell Biol.* **18**, 345-360 (2017).
7. Svergun, D. I. Determination of the Regularization Parameter in Indirect-Transform Methods Using Perceptual Criteria. *J. Appl. Cryst.* **25**, 495-503 (1992).
8. Ramsey, S., Orrell, D. & Bolouri, H. Dizzy: stochastic simulation of large-scale genetic regulatory networks. *J. Bioinform. Comput. Biol.* **3**, 415-436 (2005).
9. Hessling, M., Richter, K. & Buchner, J. Dissection of the ATP-induced conformational cycle of the molecular chaperone Hsp90. *Nat. Struct. Mol. Biol.* **16**, 287-293 (2009).
